# Advancing Cardiovascular, Neurovascular, and Renal Magnetic Resonance Imaging in Small Rodents Using Cryogenic Radiofrequency Coil Technology

**DOI:** 10.3389/fphar.2015.00255

**Published:** 2015-11-12

**Authors:** Thoralf Niendorf, Andreas Pohlmann, Henning M. Reimann, Helmar Waiczies, Eva Peper, Till Huelnhagen, Erdmann Seeliger, Adrian Schreiber, Ralph Kettritz, Klaus Strobel, Min-Chi Ku, Sonia Waiczies

**Affiliations:** ^1^Berlin Ultrahigh Field Facility, Max Delbrück Center for Molecular Medicine in the Helmholtz AssociationBerlin, Germany; ^2^German Centre for Cardiovascular ResearchBerlin, Germany; ^3^MRI.TOOLS GmbHBerlin, Germany; ^4^Center for Cardiovascular Research, Institute of Physiology, Charité—Universitätsmedizin BerlinBerlin, Germany; ^5^Clinic for Nephrology and Intensive Care Medicine, Charité Medical Faculty and Experimental and Clinical Research CenterBerlin, Germany; ^6^Bruker BioSpin MRI GmbHEttlingen, Germany

**Keywords:** magnetic resonance, MRI, cardiovascular imaging, neurovascular imaging; renal imaging, MR technology, radio frequency coils, cryogenic

## Abstract

Research in pathologies of the brain, heart and kidney have gained immensely from the plethora of studies that have helped shape new methods in magnetic resonance (MR) for characterizing preclinical disease models. Methodical probing into preclinical animal models by MR is invaluable since it allows a careful interpretation and extrapolation of data derived from these models to human disease. In this review we will focus on the applications of cryogenic radiofrequency (RF) coils in small animal MR as a means of boosting image quality (e.g., by supporting MR microscopy) and making data acquisition more efficient (e.g., by reducing measuring time); both being important constituents for thorough investigational studies on animal models of disease. This review attempts to make the (bio)medical imaging, molecular medicine, and pharmaceutical communities aware of this productive ferment and its outstanding significance for anatomical and functional MR in small rodents. The goal is to inspire a more intense interdisciplinary collaboration across the fields to further advance and progress non-invasive MR methods that ultimately support thorough (patho)physiological characterization of animal disease models. In this review, current and potential future applications for the RF coil technology in cardiovascular, neurovascular, and renal disease will be discussed.

## Introduction

For several decades animal models have served a wide span of applications in the life sciences. Transgenic systems have been invaluable for studying molecular signatures and specific cell populations as well as tools for non-invasive reporter gene imaging. Animal models that simulate human pathologies have also been indispensable for uncovering mechanisms behind major diseases as well as the identification of their respective treatments.

A thorough characterization of each animal model remains the crux of the matter. It ensures that the right conclusions are drawn from preclinical studies dealing with questions around pathogenesis and therapy as well as molecular studies that set the groundwork for future therapies and drug design. It is getting increasingly clear that most conditions and diseases, even those with an underlying genetic component, are multi-factorial and complex in nature suggesting that therapy should be equally intricate and versatile. It has been suggested that partial, but multiple, drug actions might be more efficient than a complete drug action at a single target in complex multifactorial disease. This calls for novel drug-design strategies that will depend not only on computational modeling for identifying correct multiple targets but also importantly on more-efficient and high-throughput *in vivo* testing. Further developments in non-invasive *in vivo* imaging in small rodents are necessary to guarantee this, as well as a swift and robust translation into clinical practice. For this to be achieved there is an absolute need for (i) anatomical and function imaging with a superb spatial and temporal resolution, (ii) high reproducibility in results, and (iii) longitudinal studies with sufficient statistical power.

Preclinical Magnetic Resonance Imaging (MRI) is conceptually appealing in the pursuit of basic and translational research as well as for explorations into cardiovascular, neurovascular, and renal disease. MRI has become increasingly important for small animal imaging at multiple levels of pre-clinical research. A growing number of reports manifest the advances for morphological and functional MRI of the heart, large blood vessels, CNS, and kidney. Notwithstanding its success and ubiquity, the relatively low sensitivity of conventional MRI constitutes an impediment for translational research and pre-clinical applications. Constraints common to standard room temperature RF MR detectors include contrast-to-noise-ratio (CNR) and spatial resolution but also acquisition time and signal-to-noise ratio (SNR), which are particular currencies spent for image quality.

In recent years, cryogenic RF coil technology that provides significant improvements in image quality has been made commercially available to small animal researchers. The cryogenic technology substantially increases SNR over standard room temperature RF-coils by considerably reducing thermal noise and signal losses in the RF receiver electronics. This facilitates the acquisition of high spatial resolution images within shorter scan times. The gain in SNR via cryogenic-cooling corresponds to the gain achieved by an equivalent increase in magnetic field strength, but without the extra challenges and costs—which could be prohibitive at extreme ultrahigh magnetic fields.

Recognizing the technical advancement in cryogenic RF coil technology this review attempts to make the (bio)medical imaging, molecular medicine, and pharmaceutical communities aware of this productive ferment and its outstanding significance for anatomical and functional MR in small rodents. The goal is to inspire collaborations across disciplinary boundaries and to attract basic scientists, translational researchers, clinician scientists, and new entrants into the field to advance the capabilities of non-invasive MR imaging through the RF coil technology. In the sections that follow some of the potential applications for cryogenic RF coil technology are discussed. Neurovascular applications for cryogenic RF coil technology include morphological imaging and functional brain mapping in mice. The benefits of cryogenically-cooled RF coils in supporting MRI microscopy (defined by a spatial resolution < 100 μ m) *in vivo* are demonstrated; the morphological detail reveals brain pathology in animal models of neuroinflammatory diseases, which opens the opportunity to follow neuroinflammatory processes even during the early stages of disease progression. Examples of MR angiography are presented, especially within the context of neurovascular disease. Early and frontier applications of cryogenic RF coil technology in cardiovascular MRI are surveyed together with the opportunities for high spatial resolution cardiac chamber quantification and parametric mapping; all being facilitated by the traits of cryogenic RF coil technology. Last but not least, the sensitivity gain of cryogenic RF coil technology is put to good use for renal MR microscopy and *in vivo* MRI to support explorations into renal diseases with non-invasive techniques for probing renal perfusion, hypoxia and inflammation. A concluding section ventures a glance beyond the horizon and explores future directions. Of course, MRI of small rodents is an area of vigorous ongoing research, and many potentially valuable developments will receive only brief mention here.

## Technical considerations

### State of the art

To date, cryogenic RF coils have been developed by making use of either copper or high-temperature superconducting (HTS) material. In this review we limit ourselves primarily to copper cryogenic coils (CryoProbe, Bruker Biospin, Erlangen, Germany) since these are available to a broader spectrum of users. Pioneering HTS coils have been developed by specialized research groups and are not commercially available yet. Although complex to operate, these coils can achieve SNR gains of more than 10-fold as demonstrated in small excised samples at high field (Black et al., 1993) or in the living mouse at 1.5 T (Poirier-Quinot et al., 2008).

### Image quality and signal-to-noise ratio

*Image quality* describes the perceived or quantitatively measured degradation of an image in comparison to its “perfect” counterpart. In MRI this translates into how well the reconstructed image represents the characteristics of the RF signals originating from the excited MR nuclei. Image contrasts are the core information in the majority of MRI applications. Contrasts allow the delineation of morphological structures and pathological lesions (spatial contrast) or the detection of signal intensity changes over time in functional MRI (temporal contrast). Detrimental factors for MR image quality include artifacts and noise.

The SNR is a quantitative metric of image quality with regards to noise. From an MR image SNR is commonly calculated by dividing the mean signal intensity of a uniform region of interest covering the target region by the standard deviation of the noise commonly derived from the background free of signal or artifacts. While high SNRs (>100) allow high confidence in the MR data, low SNRs (< 20) mean that the true image characteristics are increasingly masked by noise.

The SNR dependence on MR protocol parameters for a conventional 2D experiment is (McRobbie et al., 2006):

(1) $$\begin{array}{l} SNR\propto \frac{{FOV}_{\text{FE}}\cdot {FOV}_{\text{PE}}\cdot △z\cdot F_{\text{sequence}}\cdot \sqrt{NA}}{\sqrt{{BW\cdot N}_{\text{FE}}\cdot N_{\text{PE}}}} \end{array}$$

where *FOV* is the field-of-view in frequency encoding (FE) and phase encoding (PE) direction, Δ*z* is the slice thickness, *F*_sequence_ is the appropriate sequence dependent factor [including repetition time *TR* and echo time *TE* in relation to the relaxation times *T*_1_ and $T_{2}^{\left(*\right)}$], *NA* is the number of signal averages, *BW* is the receiver bandwidth across the image and *N* is the acquisition matrix size in FE and PE direction. In practice, temporal signal averaging is the most commonly used approach to balance the competing needs for high spatial resolution, high spatial coverage, temporal resolution, and sufficient signal to noise. Yet, signal averaging comes at the cost of scan time when using standard gradient echo or spin echo imaging techniques, without the implementation of acceleration techniques such as parallel imaging or compressed sensing. The square root relation of SNR with the number of averages very quickly sets a limit at which more averages can no longer be justified with a substantial SNR gain due to the severe scan time penalty governed by the power of 2 of the number of averages.

### Why cooling the radiofrequency hardware improves image quality

SNR of an MR image is closely linked to the quality of the acquired RF signals it is composed of. These RF signal measurements—like all analog electronic measurements—are degraded by noise, which reduces signal quality and hence image quality. RF coil efficiency relies on overcoming thermal noise induced by conductive samples (Hoult and Lauterbur, 1979). The noise in MR signal acquisitions originates predominantly from thermal (Brownian) motion of electrical charge carriers within the passive receiver electronics (e.g., coil, conductors, passive components) and within the sample itself. Particularly susceptible to noise are those parts of the electronics in which the RF signal is very small, i.e., from the RF coil until the preamplifier output. The contribution of a 50 Ohm preamplifier to the noise cannot be so easily described because of the reactive part of its impedance, though it can be evaluated by acquiring and comparing noise images at different temperatures e.g., at 77 and 293 K (Poirier-Quinot et al., 2008; Vaughan and Griffiths, 2012).

The concept of cooling RF coil hardware to reduce thermal noise was originally proposed in the 1970s by D. I. Hoult and R. E. Richards (Hoult and Richards, 1976), although the first actual application of cooled RF coils in room-temperature samples was introduced nearly a decade later (Styles et al., 1984). The temperature dependency of the SNR can be described with (Kovacs et al., 2005; Junge, 2012):

(2) $$\begin{array}{l} SNR\propto \frac{\omega_{0}B_{1}}{\sqrt{R_{S}T_{S}+R_{C}T_{C}+\left(R_{S}+R_{C}\right)T_{A}}} \end{array}$$

where ω_0_ is the Larmor angular frequency, *B*_1_ is the effective RF field in the sample volume, *R*_*S*_ represents the so-called magnetic and electric loss in the sample (sample resistance), *R*_*C*_ is the resistive loss in the RF coil and *T*_*S*∕*C*∕*A*_ is the temperature of the sample, coil, and the noise temperature of the preamplifier, respectively. Cooling reduces the coil noise *R*_*C*_*T*_*C*_ and preamplifier noise (*R*_*S*_+ *R*_*C*_)*T*_*A*_ and directly increases SNR. An additional benefit of cooling the receiver electronics is owed to the decrease of electrical resistances in the conductor (*R*_*C*_) with decreasing temperatures. Reducing losses within the RF receive chain enhances the SNR.

However, SNR is also dependent on other factors such as frequency ω_0_, RF coil geometry/quality (creating the effective *B*_1_) and sample noise *R*_*S*_*T*_*S*_. Comparing the impacts of coil noise and sample noise on SNR in the context of RF coil size helps to answer the question whether—and under which circumstances—cooling the RF coil is worthwhile. As shown by Junge (2012), the coil and sample noise contributions to SNR at room temperature are of comparable magnitude for a single loop coil with a diameter of approximately 3.0 cm (at 1.5 T) or 1.4 cm (at 9.4 T). For larger RF coils sizes, as commonly used in human MRI, sample noise contributions dominate SNR. For small animal MRI the volumes of interest and RF coil sizes are typically in the range of 1–2 cm, rendering coil noise contributions comparable or larger than sample noise contributions. Although cooling the RF hardware will always reduce noise, the engineering and cost of cryogenic RF hardware starts to pay off only when the thermal noise originating from the receiver electronics is comparable or larger than the noise originating from the sample (patient, animal, object under investigation). It is for that reason that cryogenic RF coils have been developed primarily for nuclear magnetic resonance spectroscopy (NMR; see review Kovacs et al., 2005) using small samples of few millimeter diameter or few cubic-millimeter volume and more recently for small animal MRI.

The theoretical upper limit for the SNR improvement at a magnetic field strength of 9.4 T (f = 400 MHz) is an estimated factor of 2.8 for a transmission line resonator consisting of two planar split rings (with an inner diameter of 14 mm) that are placed on either side of a dielectric substrate, under the assumption of cooling the RF coil from room temperature down to 0 K (Junge, 2012). Cooling the preamplifier has a similar effect on SNR for small samples (Kovacs et al., 2005), but taking a typical preamplifier noise temperature of 15 K with a dominating sample noise (*R*_*S*_ >> *R*_*C*_) at 310 K, the SNR equation shows that the noise contribution from the preamplifier is not that critical. In practice however the SNR gain achievable by a cryogenic RF coil is lower than the theoretical estimate. The RF hardware cannot be cooled down to 0 K. RF coil and preamplifier are typically operated at temperatures around −253°C (20 K) and −193°C (80 K), respectively. Notwithstanding these limitations, an SNR gain as high as 2.5 was reported for *in vivo* mouse brain MRI when comparing cryogenic RF hardware (400 MHz CryoProbe, Bruker Biospin MRI GmbH, Ettlingen, Germany) with a conventional room temperature RF coil setup of similar geometry (Baltes et al., 2009).

According to Hoult and Lauterbur (1979), sample resistance *R*_*S*_ increases quadratically with the Lamor frequency ω_0_ for a well-designed RF coil. Thus, it may counterbalance the upper frequency term in the above Equation (2) toward higher frequencies. Higher SNR gains are expected at frequencies lower than 400 MHz and have been indeed reported at 200 MHz using the same cooled copper coil technology (Ratering et al., 2008). The gain becomes even higher when using the HTS coil technology at lower frequencies (Darrasse and Ginefri, 2003): SNR gains larger than 10-fold were achieved at 64 MHz in the living mouse brain (Poirier-Quinot et al., 2008). Thus, the cryogenic technology will be particularly valuable for preclinical studies when used in combination with clinical field strengths.

### Cryogenic radiofrequency coil hardware

The first commercial cryogenically cooled RF probe for NMR spectroscopy was installed in 1999 (Kovacs et al., 2005). It took almost 10 years for this technology to mature and to become commercially available for small animal MR scanners. The feasibility and benefit of cryogenically-cooled MRI RF probes was demonstrated for *in vivo* mouse brain imaging at 9.4 T (Baltes et al., 2009). This success built upon the pioneering explorations into cryogenic RF coil designs for biomedical MRI by researches such as Hurlston et al. (1999), Ginefri et al. (2007), Nouls et al. (2008) and the successful development of a prototype for 4.7 T (Ratering et al., 2008).

Modern cryogenic RF hardware employs high temperature superconducting (HTS) materials to dramatically reduce resistive losses (Black et al., 1993; Hurlston et al., 1999; Darrasse and Ginefri, 2003; Ginefri et al., 2007; Nouls et al., 2008; Junge, 2012; not commercially available yet). Cold helium gas has replaced liquid cryogens as cooling media. Simply speaking, Helium gas is compressed in one chamber and then chilled through expansion in another chamber in a closed-loop cooling system—comprised of the RF coil, a cryogenic cooling unit and a helium compressor. The waste heat produced during the gas compression is transferred from the helium compressor to a cold water system via a connected water chiller.

**Figure 1:**
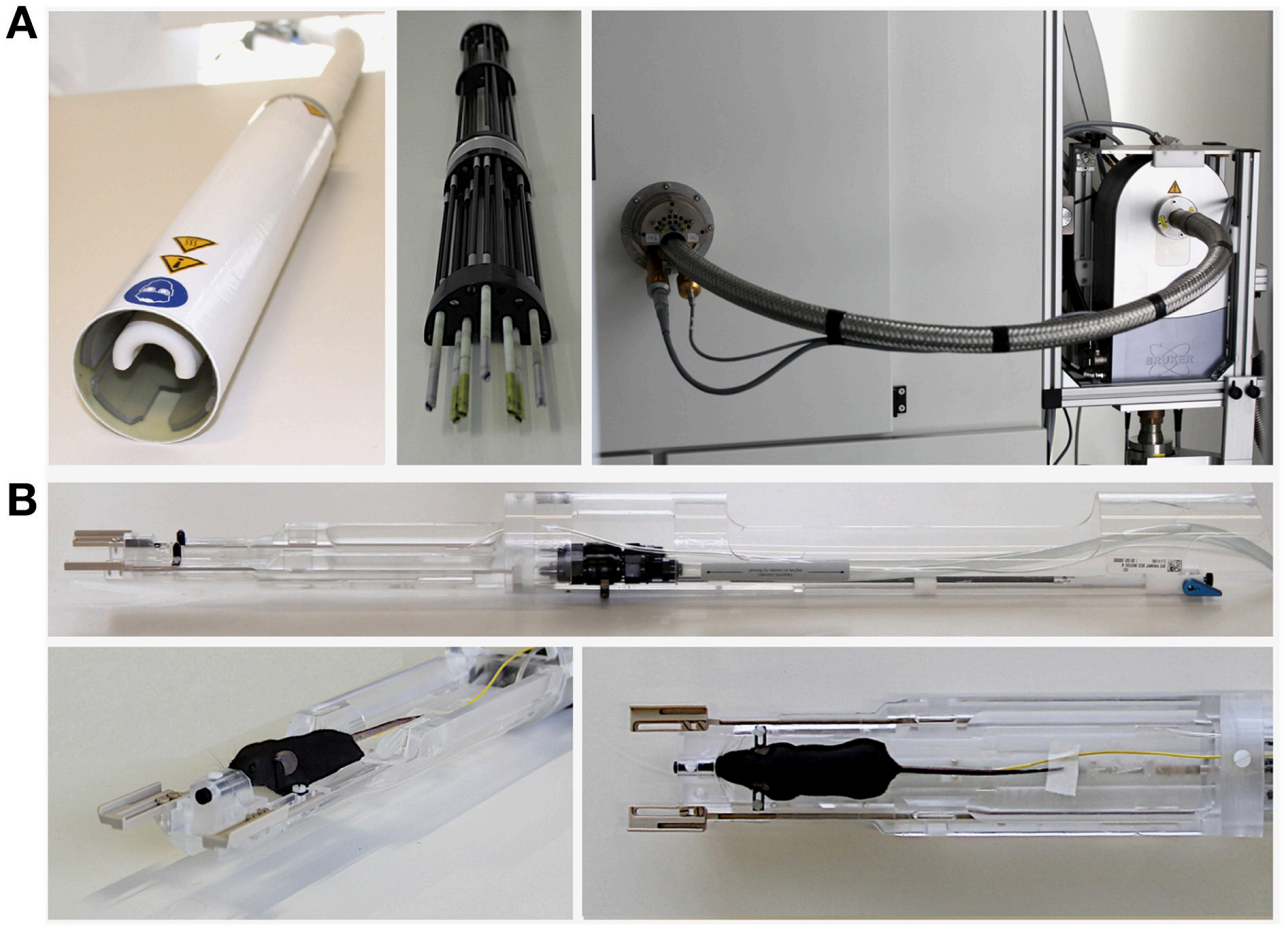
**Hardware components and setup of MRI with cryogenic radiofrequency coil technology (9.4 T MRI system 94/20 Biospec with 400 MHz Quadrature TxRx CryoProbe, Bruker Biospin MRI GmbH, Ettlingen, Germany; some parts not shown)**. **(A)** left: RF coil head (CryoProbe); center: tuning/matching unit that is attached to the rear of the CryoProbe when installed in the scanner; right: view of the rear of the magnet with the CryoProbe and tuning/matching unit inserted into the magnet bore, which are connected to the cryogenic preamplifier visible on the right hand side. **(B)** top: cradle tailored to the CryoProbe, incorporating a warm water based floor heating, nose cone with tooth bar (black), and outlet of anesthetic gas, ear bars (black) for fixation of the mouse head, spacers (beige) to adjust the z-axis position of the cradle underneath the CryoProbe, a lever system to permit lifting the cradle slightly upwards closer to the CryoProbe once the cradle has been fully inserted and the mouse is located below the CryoProbe; bottom left and right: front of the cradle with a mouse set up for MRI, showing the muzzle of the mouse in the nose cone, the ear bars fixing the head (see view from above in right panel) and a rectal temperature probe (yellow) to monitor core body temperature.

For a review of cryogenically cooled RF coil hardware, we will focus here on the range of commercially available cryogenic RF surface coils for preclinical MRI (CryoProbe™, Bruker Biospin MRI GmbH, Ettlingen, Germany). The RF coil head (CryoProbe; Figure 1A left) is installed at the center of the magnet bore. In this type, a set of torque rods (Figure 1A center) are fitted to the RF probe to allow its tuning and matching from the rear end of the MR system. The preamplifier and the interconnection between preamplifier and CryoProbe are also cryogenically cooled (Figure 1A right). Positioning of the mouse underneath the CryoProbe is facilitated by a dedicated animal cradle (Figure 1B top), which features a warm water heated floor, nose cone with tooth bar and anesthetic gas outlet, ear bars for head fixation, and spacers to adjust the z-axis position of the cradle, as well as a lever system to permit lifting the cradle closer to the CryoProbe once both are fully inserted and the mouse is located below the RF coil (Figure 1B bottom).

Since the introduction of the first cryogenic MRI RF coil the range of CryoProbes is steadily expanding. The initial mouse brain quadrature transmit/receive MRI CryoProbes (for 4.7–15.2 T MR systems) are now-a-days complemented by four-channel array receive-only CryoProbes (for 7.0 and 9.4 T MR systems), four-channel array CryoProbes for rats, and mouse X-nuclei CryoProbes (for 9.4 T; e.g., ^13^C, Sack et al., 2014). The latter are used in conjunction with a built-in room temperature ^1^ H RF coil for decoupling and acquisition of anatomical reference images. Finally, the receive-only CryoProbes allow for the use of a room temperature volume resonator for RF excitation, which improves transmission field (${\text{B}}_{1}^{+}$) homogeneity.

### Characteristics of cryogenic RF surface coils—the pros and cons

**Figure 2:**
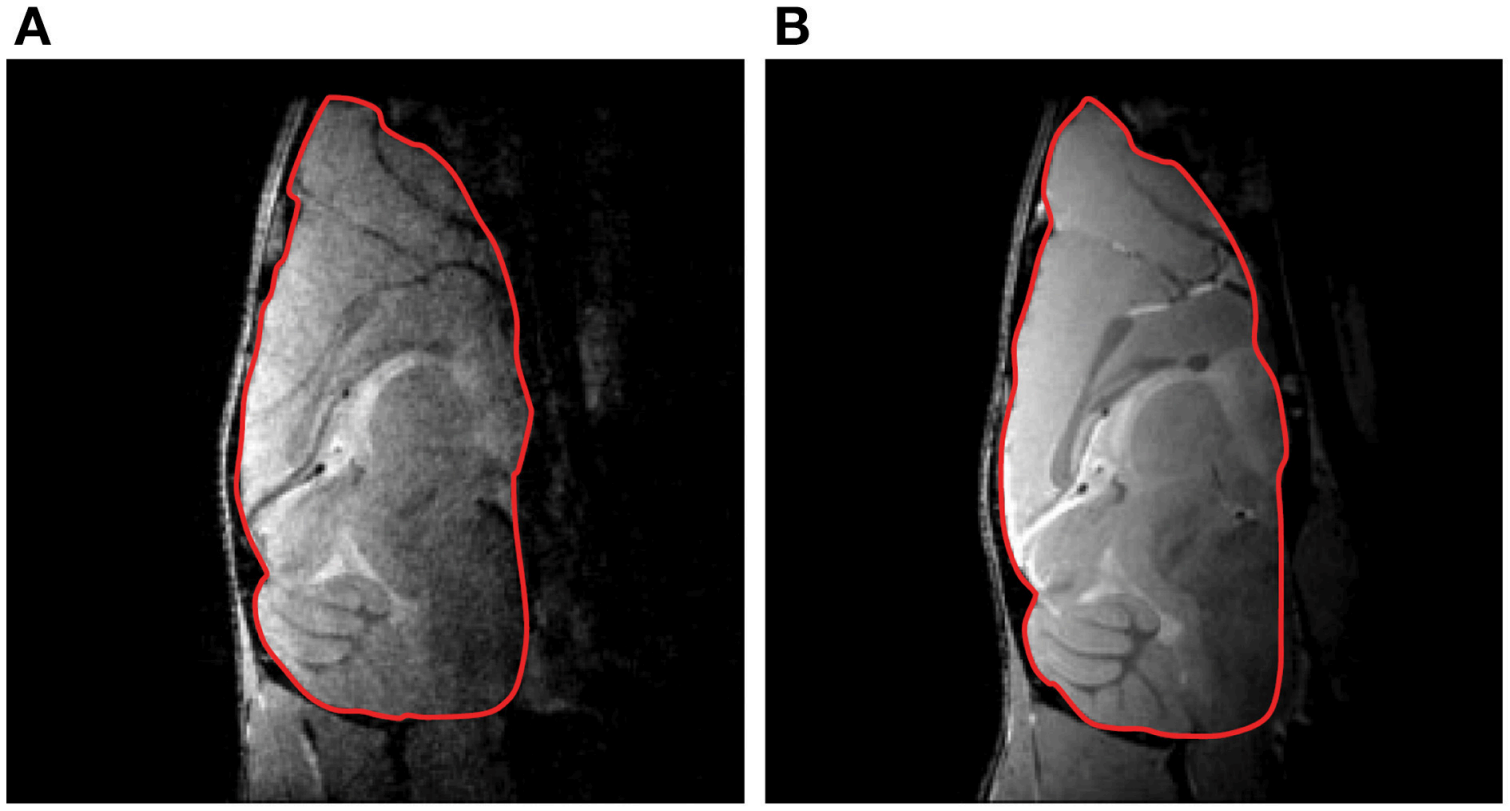
**Mouse brain gradient-echo images acquired at 9.4 T with a RT receive-only RF coil (A) and a transmit/receive quadrature CryoProbe (B)**. The overall SNR gain for the delineated brain region of interest was 2.8. From Junge (2012), by permission of John Wiley & Sons Limited.

Cryogenic cooling of the RF coil and preamplifier more than doubles the SNR compared with an equivalent RT coil setup. *In vivo* mouse studies revealed typical SNR gains of 2.5–2.8 in the brain (Figure 2; Baltes et al., 2009, 2011; Junge, 2012), 3.0–5.0 in the heart (Wagenhaus et al., 2012), and 3.0–3.5 for ^13^C spectroscopy in the brain (Sack et al., 2014). These examples of sensitivity enhancements obtained for 400 MHz (9.4 T) may only serve as a basic reference because the SNR gain depends on the frequency (see equation for SNR and Ratering et al., 2008) and the choice of RT coil against which the performance of the CryoProbe is benchmarked.

In practical terms, a 100% SNR gain by use of a CryoProbe is substantial enough to facilitate an improvement in spatial resolution from e.g., (100 × 100) μm^2^ to (71 × 71) μm^2^ by reducing the FOV. Maintaining the same FOV and acquisition time the spatial resolution can be improved to (63 × 63) μm^2^, as a result of increasing the matrix size (by 58%) combined with a reduction in the number of averages (by 36%). Alternatively an acquisition time reduction by 75% could be attained with this SNR gain via reducing the number of averages.

An SNR gain of 100% is equivalent to significantly increasing the magnet field strength, which is not only more cost intensive, but also comes along with challenges such as increased susceptibility artifacts, disadvantageous relaxation time changes, extra constraints for RF coil design at higher frequencies due to wave length shortening and adverse effects for physiological signals due to the interference with the magnetic field.

**Figure 3:**
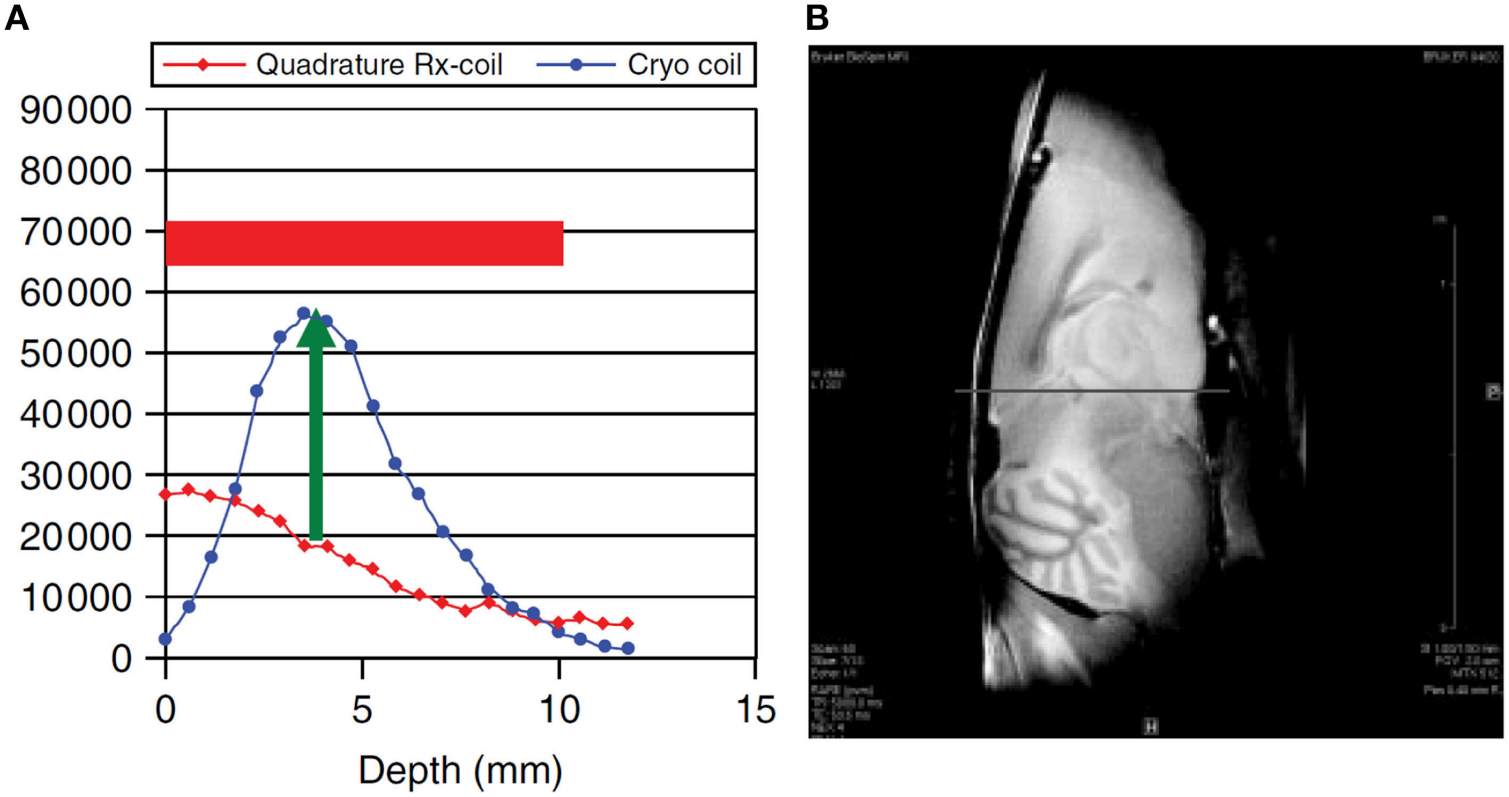
**SNR dependence on distance from RF coil surface (A) and corresponding *ex vivo* MR image of a mouse brain (B)**. Comparison of a 400 MHz transmit/receive linear CryoProbe with a RT quadrature receive-only RF coil. From Junge (2012), by permission of John Wiley & Sons Limited.

**Figure 4:**
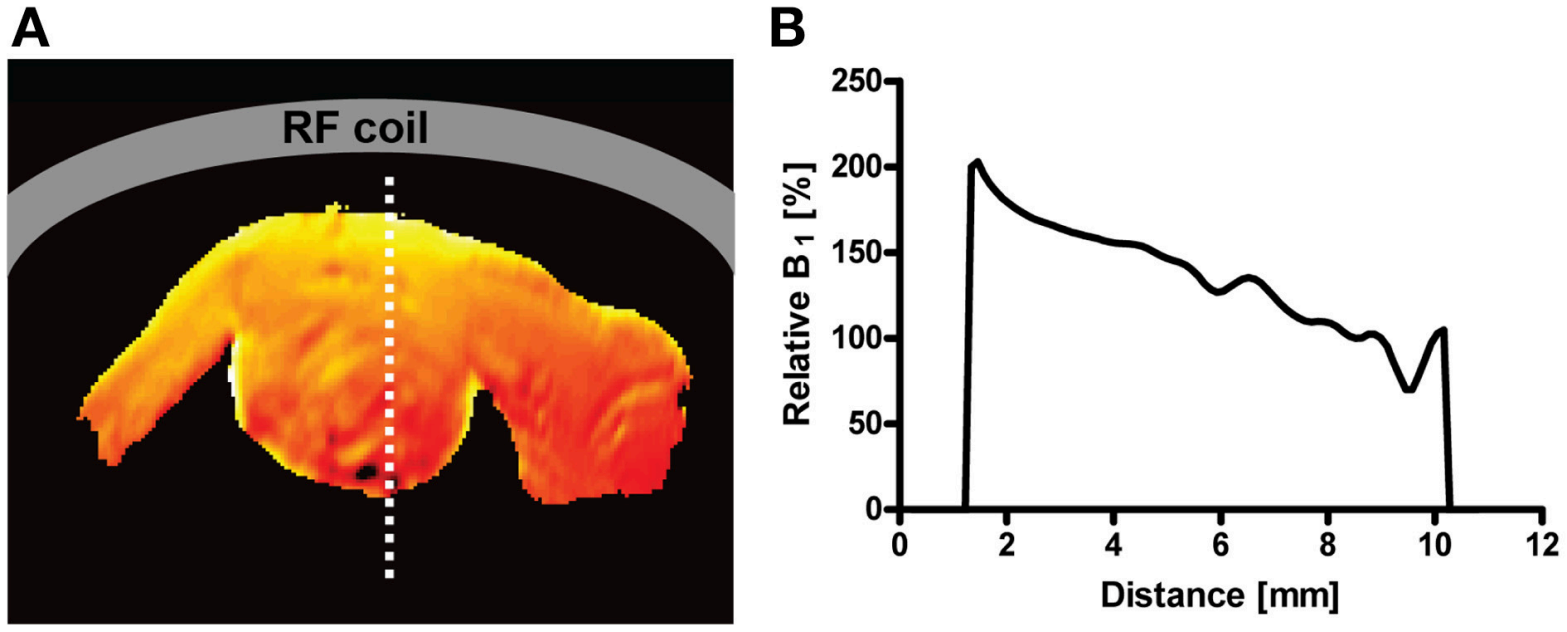
**(A):** illustration of the spatial variation of flip angle as relative ${\text{B}}_{1}^{+}$-map of a mouse heart in short axis view for a 400 MHz transceiver CryoProbe with surface coil design. **(B)**: Plot of the ${\text{B}}_{1}^{+}$ profile along a line crossing the heart (dotted line in ${\text{B}}_{1}^{+}$-map). The ${\text{B}}_{1}^{+}$ decrease from anterior (close to RF coil) to posterior is approximately 50% (factor of 2). From Wagenhaus et al. (2012), by permission of Public Library of Science.

A surface coil design CryoProbe is essential to position the coil as close as possible to the object under investigation, to conform the coil geometry to the target anatomy, to keep coil size small and remain within the coil noise dominated regime. Although beneficial for signal sensitivity, an inherent limitation of surface coils is an inhomogeneous distribution in both the transmit RF field (${\text{B}}_{1}^{+}$) and receive sensitivity profile (${\text{B}}_{1}^{-}$). Figure 3 demonstrates the depth dependence of SNR for a spin-echo imaging protocol. SNR is greatly enhanced by the CryoProbe, but only within a certain range of depth, here approximately 2–8 mm from the surface of the coil. The receive sensitivity profile of the surface RF coil reduces the ability to detect RF signals from locations very close to as well as far away from the RF coil. These variations might be a result of a transmit RF field inhomogeneity of the coil which then translates into a variation in flip angle across the field of view, thereby impacting on the signal intensity and T_1_ image contrast. This applies to flip angles of excitation pulses as well as any other RF pulses, such as refocussing RF pulses with the exception of adiabatic pulses. Over a typical field of view of 6–8 mm (perpendicular to RF coil surface) the relative B_1_ that is proportional to the flip angle can vary by up to a factor of 2 (Figure 4; Baltes et al., 2009; Wagenhaus et al., 2012). In gradient-echo images this variability may often go unnoticed. However, in spin-echo images, large deviations from the 90° excitation pulses and the 180° refocusing pulses will eventually lead to inevitable signal losses. Transmit/receive surface coils like most of the CryoProbes require RF power adjustment on a coronal slice (rather than the standard axial slice) which must be carefully positioned to achieve sufficient RF power at larger depths while avoiding signal loss close to the RF coil in spin-echo acquisitions due to too much RF power. ${\text{B}}_{1}^{-}$ inhomogeneity can be largely avoided with the recently available receive-only CryoProbes, which make use of an additional RT volume resonator for RF transmission.

In conclusion, various factors play a role in determining the actual SNR gain, including magnet field strength, MR nucleus, distance to the RF coil, RF power adjustment as well as the MRI acquisition method. The typical improvement in SNR by commercially-available CryoProbes can be expected to be a factor of 2–3, which can be translated into more than 60% higher spatial in-plane resolution or 75% shorter acquisition time.

## Neurovascular applications

### Animal models of multiple sclerosis

The two key benefits of an SNR gain when using cryogenically-cooled coils—namely reduced scan time and/or increased image detail—are fundamental for studying dynamic pathological processes in animal models of disease. In several pathological conditions, especially those related to inflammation and vascular remodeling, an important challenge is to differentiate between hemodynamic alterations, inflammation, and degenerative processes during different stages of disease. Differentiating between pathological processes is essential in chronic neuroinflammatory conditions such as multiple sclerosis (MS) that include an inflammatory, demyelinating and neurodegenerative component. A differentiation between these processes is necessary to make the right therapeutic decision and follow the correct line of treatment for each individual patient (Sinnecker et al., 2012a,b, 2013; Wuerfel et al., 2012; Kuchling et al., 2014).

The histopathological hallmark of MS is the demyelinated plaque, which is associated with perivascular and parenchymal inflammatory cell infiltration and axonal injury (Kuhlmann et al., 2008). MS plaques can occur throughout the CNS; in periventricular and deep white matter, optic nerves and tracts, cerebellar peduncles, brainstem, spinal cord, and also in the gray matter (Sinnecker et al., 2012b). MRI is the most sensitive test to detect and demonstrate MS lesions (Milo and Miller, 2014): while active inflammation is associated with newly appearing, hyperintense MS lesions on T_2_-weighted MRI and enhancement on T_1_-weighted MRI after contrast agent (Barkhof et al., 1997; Brück et al., 1997), neurodegeneration is associated with hypointensities on T_1_-weighted images and indicates severe tissue damage (Van Waesberghe et al., 1999).

An animal model that resembles MS pathology is the experimental autoimmune encephalomyelitis (EAE). EAE can be induced by immunizing different susceptible species of animals with specific CNS antigen e.g., proteolipid protein (PLP) in SJL/J mice. The EAE model has been indispensable for understanding neuroinflammatory disease (Ransohoff, 2009) and for evaluating the effectiveness of nascent therapeutic approaches for MS (Ben Nun et al., 1981; Steinman and Zamvil, 2006; Ransohoff, 2012). Similarly to MS, lesions that are hyperintense on T_2_-weighted MRI are present in EAE brains and correspond to inflammation, demyelination and neurodegeneration (Deboy et al., 2007). Also similar to MS, EAE lesions are distributed in time and space (Baxter, 2007), commonly in the brain stem, midbrain, cerebellum and periventricular area (especially in non-human primates) but also in spinal cord, corpus callosum and cerebral gray matter in small rodents (Verhoye et al., 1996; Hart et al., 1998; Boretius et al., 2006; Deboy et al., 2007; Wuerfel et al., 2007; Waiczies et al., 2012).

**Figure 5:**
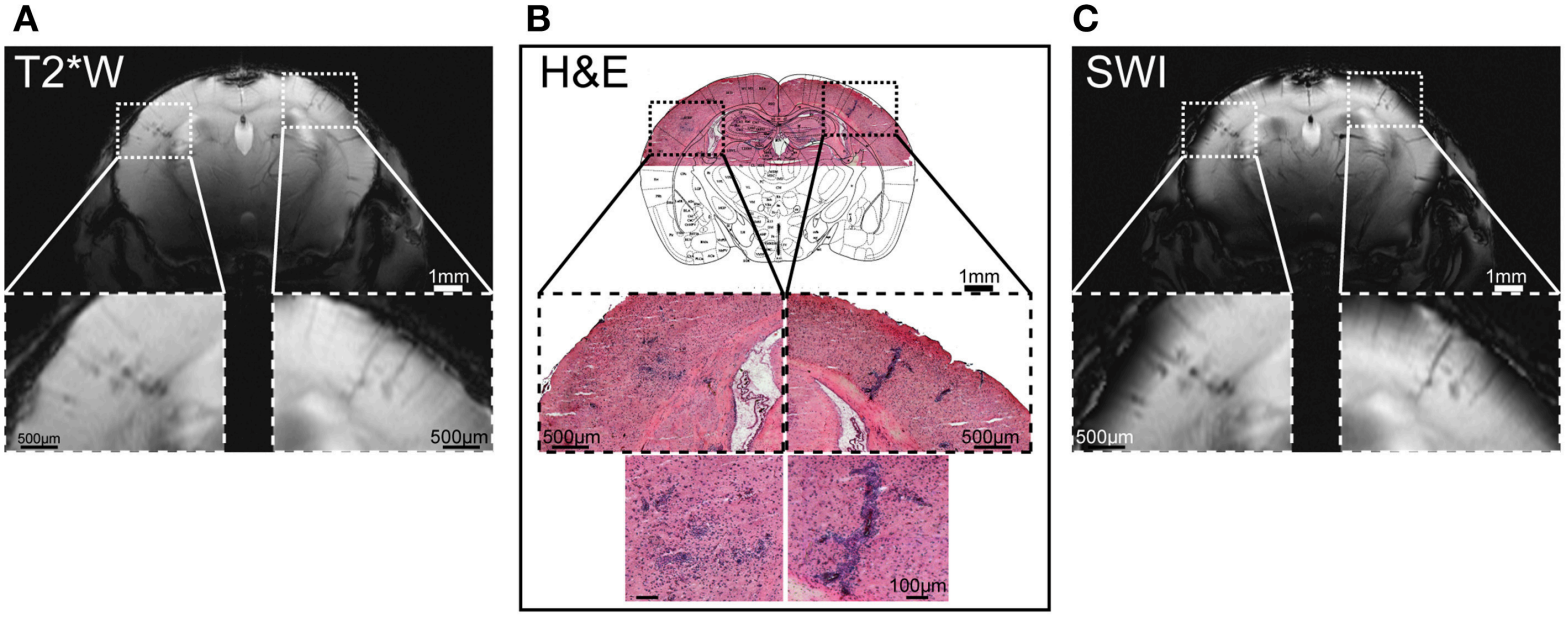
**Hypo-intense regions on microscopic MRI correspond to cellular infiltrates detected by histology**. **(A)** Coronal T_2_* weighted imaging (T_2_*W) using a multislice fast low angle shot (2D FLASH: TR/TE: 473/18 ms, FA 40°, matrix 512 × 512) sequence with an in plane resolution of (35 × 35) μm^2^, 22 slices of 500 μm, acquisition time to image the whole mouse brain = 11 min. **(B)** Cellular infiltrates in cerebral cortex; the overview of the H&E histology is overlaid with a coronal slice (plate 41) from Paxinos and Franklin (2013), by permission of Elsevier. **(C)** Susceptibility weighted imaging (SWI) of T_2_*W scans using fully-automated post-processing by ParaVision 5.1 (Bruker, Ettlingen, Germany). From Waiczies et al. (2012), by permission of Public Library of Science.

Brain MR microscopy is an invaluable tool to visualize succinct inflammatory patterns, even prior to neurological disease in the EAE. The main strengths of cryogenically-cooled coils to boost SNR and thereby provide high resolution MR microscopy and/or reduced scan time are best appreciated in animal models such as EAE that undergo highly dynamic disease profiles. Apart from the established hyperintense lesions on T_2_-weighted images (commonly in the cerebellum), focal hypo-intense lesions were identified in the somatosensory cortex of EAE mice, when using high spatial resolution T_2_^*^ and T_2_ MRI in association with the CryoProbe (Waiczies et al., 2012). With the cryogenic system, an in plane resolution is as good as (35 × 35) μm^2^ and complete coverage of the brain could be achieved on both T_2_^*^ and T_2_-weighted imaging. With this capacity clear punctate lesions in cerebral gray matter could be revealed in association with intracortical vessels and distributing into the corpus callosum (Waiczies et al., 2012). Thanks to the spatial resolution facilitated by Cryoprobe technology the lesions that were revealed *in vivo* by MR histology could be clearly corroborated as inflammatory infiltrates with hematoxylin and eosin histology (Figure 5).

To gain a comprehensive and longitudinal view of brain inflammation, particularly during the early stages of EAE, MR methods that increase SNR are advantageous not only to increase detail via high resolved brain microscopic imaging but also to reduce scan time. Highly resolved MR images of the whole brain could be achieved in 11 min with the CryoProbe (Figure 5; Waiczies et al., 2012). By reducing scan time, the number of measurements per individual animal can be increased during progression of disease. Repeated MR measurements are fundamental when making assessments regarding structural changes relevant to the pathology; even macroscopic changes (that do not require highly resolved MRI to be revealed) can be easily revealed. As a result of the reduced scan time and repeated measurements achievable by the CryoProbe, ventriculomegaly prior to neurological symptoms was revealed in the EAE (Lepore et al., 2013).

**Figure 6:**
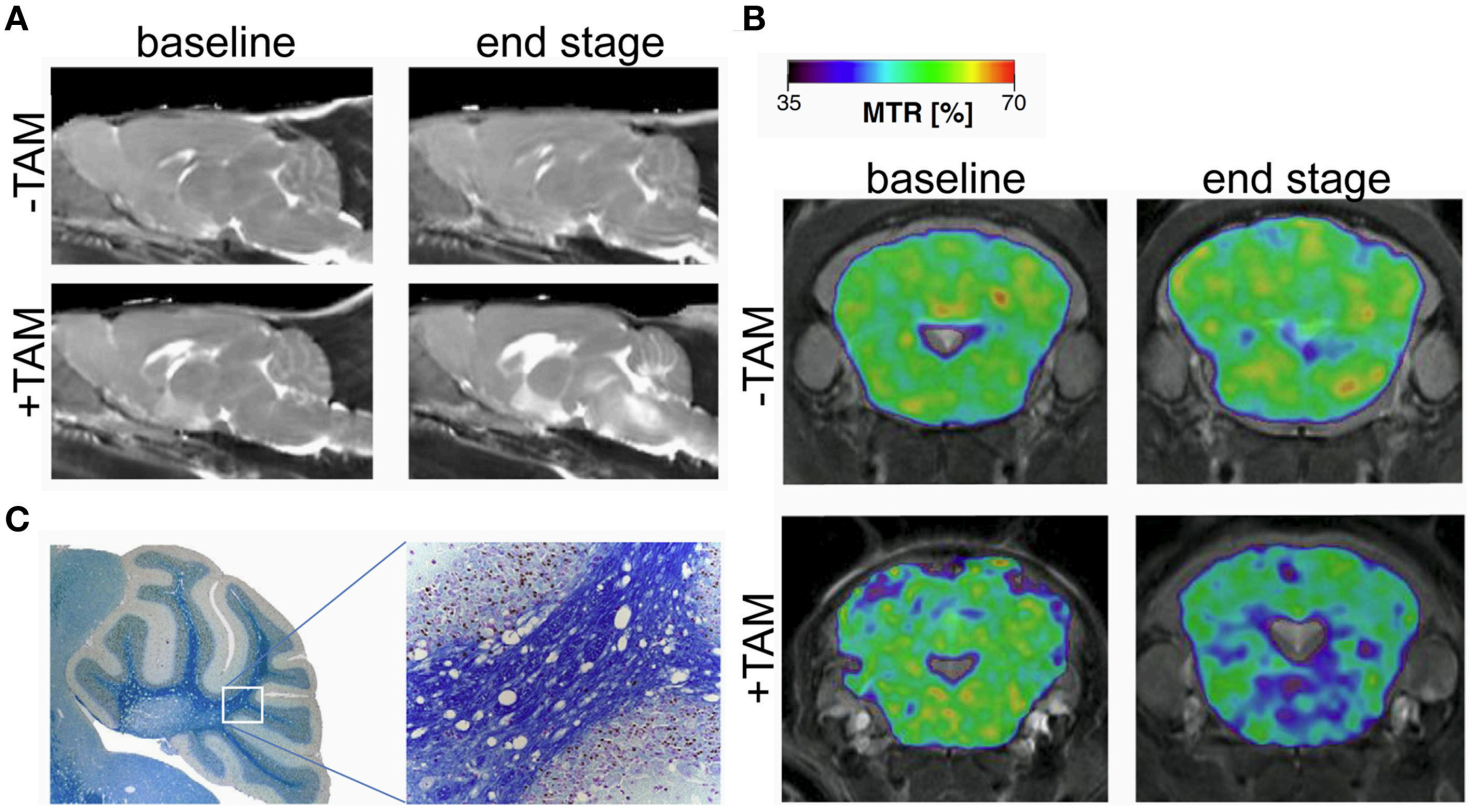
**MRI signature in a novel mouse model of genetically induced adult oligodendrocyte cell death**. **(A)** Parasagittal, quantitative T_2_ maps at baseline (day 2), and end stage (day 41) oligodendrogliopathy following tamoxifen-induced ablation of oligodendrocytes (+TAM) and controls (−TAM). **(B)** MTR maps from representative animals before (−TAM) and after (+TAM) oligodendrocyte ablation at baseline (day 2) and end stage (day 41). MTR maps are color-coded for percentage and superimposed on T_2_-weighted images (cerebellum and brainstem section at the level of −2.3 mm relative to Bregma). **(C)** Magnifications of Histological cerebellar sections from end stage diseased animal stained with Luxol-Nissl for myelin content. From Mueggler et al. (2012), by permission of Academic Press.

In the past few years animal models that simulate the neurodegenerative and demyelinating components of MS have been studied independently of the inflammatory component (Ransohoff, 2012). For instance transgenic models involving suicide genes that induce oligodendrogliopathy are powerful tools for studying non-inflammatory demyelination and remyelination (Traka et al., 2010; Pohl et al., 2011). Using a cryogenically-cooled RF coil, pronounced T_2_ hyperintensities were revealed in brain stem and cerebellar structures of this model (Figure 6A); these changes were accompanied with a decreased magnetization transfer ratio (MTR; Figure 6B; Mueggler et al., 2012). MTR reflects the exchange of magnetization between pools of differently mobilized protons (Wolff and Balaban, 1989), commonly a rather freely mobile pool and a rather immobile pool associated with macromolecules, such as in axonal membranes or myelin (Alonso-Ortiz et al., 2015). MTR correlates significantly with the degree of myelination as shown in postmortem tissue of MS patients (Schmierer et al., 2004). The decline in MTR that was observed in the oligodendrogliopathy model with the aid of the CryoProbe (Figure 6B) could be corroborated with evidence of white matter pathology upon histological analysis (Figure 6C; Mueggler et al., 2012).

### Animal models of cerebral amyloid angiopathies

**Figure 7:**
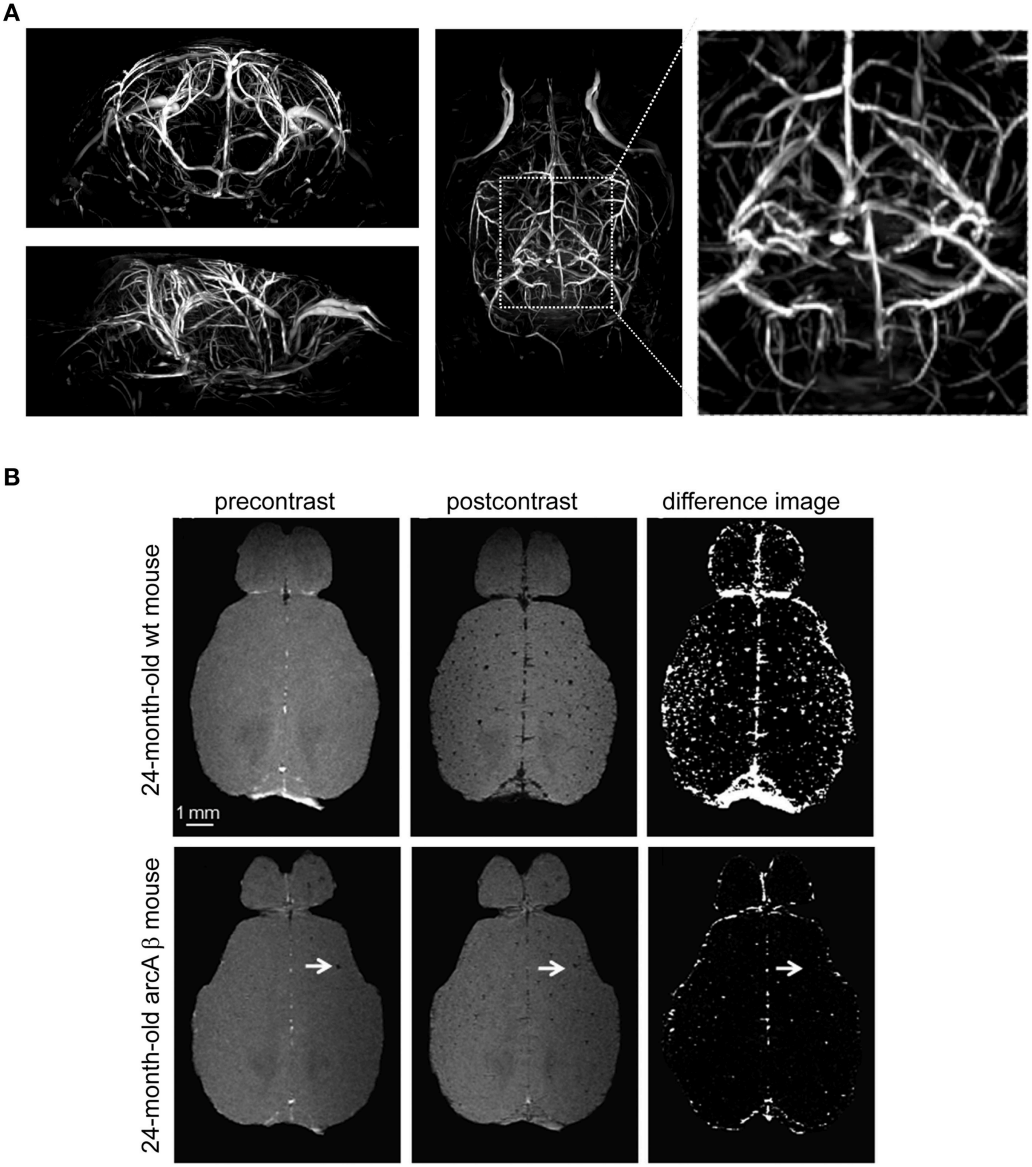
**Visualization of vascular structures using cryogenically cooled RF coil technology to provide high resolution time-of-flight (TOF)-MRA and spin dephasing following intravascular iron oxide contrast agent**. **(A)** High-resolution 3D TOF-MRA (axial and horizontal view) of the intracranial and extracranial vasculature of a 2-month-old wild type control mouse using a gradient echo sequence with flow compensation acquired at 9.4 T using the CryoProbe (TR = 30 ms, TE = 5.9 ms, FOV = (30 × 15 × 15) mm^3^, 512 × 256 × 256, isotropic resolution = 59 μm^3^. From Supplementary Data in Waiczies et al. (2012), by permission of Public Library of Science. **(B)** Contrast-enhanced cerebral MRA of a 24-month-old wt mouse and an age-matched arcAβ mouse before and after administration of a superparamagnetic iron oxide contrast agent. Images were acquired using a 3D FLASH sequence [TR = 150 ms, TE = 2.9 ms, FA = 20°, FOV = (15 × 12 × 2.2) mm^3^, spatial resolution of (60 × 60 × 61) mm^3^]. Difference images were obtained by subtraction of postcontrast image from precontrast image. The arrows point to focal hypointense areas that are present before administration of the contrast agent. Scale bar: 1 mm. From Klohs et al. (2012), by permission of Elsevier.

Neurodegenerative pathologies that involve cerebrovascular dysfunction include the cerebral amyloid angiopathies (CAA); these form a major part of the pathophysiology of Alzheimer's disease (AD; Biffi and Greenberg, 2011). Also in this field, transgenic systems and high resolution MR imaging are proving extremely valuable for investigating the role of vascular dysfunction and amyloid deposition in the pathophysiology and treatment of CAA and AD (Klohs et al., 2014). An important role for MR methods is for them to capture vascular remodeling as a result of hemodynamic alterations; this is important when studying the outcome of CAA on vascular integrity and function (Salat, 2014). The cerebral vascular tree can be assessed by different magnetic resonance angiography (MRA) techniques. The signal in time-of-flight (TOF)-MRA depends on the blood flow and is useful for determining changes in the architecture of major blood vessels as well as reduced blood flow in mouse models of AD and CAA. When spatial resolution is limited, the tissue surrounding vessels also contributes to the MR signal. Originally TOF-MRA was limited to large cerebral vessels with high flow rates; small vessels suffer from both spin-saturation artifacts and partial volume effects and their edges are more difficult to visualize (Lin et al., 1997). The application of a cryogenically-cooled coil system to increase the resolution of TOF-MRA is a key to improve the detection of smaller vessel (Figure 7A) in order to help identification of pathologies related to neurovascular disease. The enhanced spatial resolution per unit time introduced by the cryogenic system is key to start overcoming some of the partial volume effects that contribute to loss of small vessel visualization. Using a spatial resolution of (150 × 100 × 100) μm^3^, flow disturbances at the level of the circle of Willis could be observed in the major arteries of aged animals containing the amyloid precursor protein (APP)23 transgene using TOF-MRA (Beckmann et al., 2003). Administration of superparamagnetic iron oxide (SPIO) particles in association with signal attenuations on three-dimensional (3D) gradient-echo MRI also revealed CAA-related microvascular lesions in different transgenic mouse models of AD including APP23 even at a spatial resolution of (109 × 133 × 300) μm^3^ (Beckmann et al., 2011). Using a cryogenically-cooled coil system and a spatial resolution of (60 × 60 × 61) μm^3^, contrast-enhanced (CE) micro (μ)MRA identified age-dependent and CAA-related remodeling of the cerebral microvasculature in arcAβ mice (Klohs et al., 2012). These transgenic mice express human APP and are characterized by strong CAA pathology (Merlini et al., 2011). Using longer san times, CE-μMRA was performed at even higher spatial resolutions of (31 × 31 × 93) μm^3^ and vessels could be tracked far into the periphery (Figueiredo et al., 2012). Contrast enhanced (CE)-μMRI is expected to complement TOF-MRA (Klohs et al., 2012); after administration of the iron oxide contrast agent, hypointensities not discernable on the precontrast image become visible; these represent intact blood vessels (Figure 7B). In this study, CE-μMRA in association with a CryoProbe revealed a reduction in the density of the microvasculature in the arcAβ mouse during advanced disease state (Klohs et al., 2012).

### Tumor models

The summed-up advantages of the Cryoprobe are of significant benefit to visualize the pathology of tumor disease as well as assess the outcome of treatment options. One major drawback of standard clinical tumor imaging is associated with limitations in spatial resolution. This major limitation hinders an adequate delineation of tumor tissue from the surrounding healthy tissue prior to surgical tumor resection and results in the reported brain shift during navigated neurosurgical procedures (Reinges et al., 2004). A thorough delineation of tumor borders could be recently achieved in an animal model of high grade glioma by using a Cryoprobe for acquisition of MR images with an in-plane spatial resolution of 51 mm (Ku et al., 2013; Vinnakota et al., 2013). In these studies tumor MR microscopy was employed to determine the molecular mechanisms behind tumorgenesis as a precursor to therapeutic studies directed toward identified molecular targets.

Angiogenesis and neovascularization are histopathological hallmarks of cancer (Welti et al., 2013). Molecular determinants that pave these processes need to be targeted to overcome the current limitation of standard treatment regimen in reaching malignant gliomas (Agarwal et al., 2011). The tumor vasculature is functionally and structurally irregular and anti-angiogenic therapies aim to normalize this vascular irregularity (Jain, 2005). MR methods measuring perfusion, permeability, and vessel size has been a necessary tool, commonly requiring application of different contrast agents to monitor tumor neovascularization especially as a means of assessing antiangiogenic therapy response (Zwick et al., 2009; Knutsson et al., 2010). Recently, vessel architectural imaging (VAI) was introduced and exploited to study tumor vessel caliber, hemodynamics, and relative oxygen saturation and used as a determinant of vessel type and function, especially for therapy response, in glioma patients (Emblem et al., 2013). Vessel size imaging (VSI) and DCE-MRI produce complementary information about the tumor vasculature but need to be performed separately due to the interaction between contrast agents regarding magnetization (Beaumont et al., 2009; Zwick et al., 2009). Recently, a cryogenically-cooled RF coil system was used to combine VSI and DCE-MRI in a glioma mouse model; a single shot gradient echo spin echo (GE-SE) sequence was combined with echo-planar imaging (EPI) and one contrast agent was used (Kording et al., 2014). The superior SNR of the cryogenically-cooled coil that made an increase in the receiver bandwidth possible, in combination with careful shimming routines, ascertained that segmentation and geometry between T_2_ maps, ADC maps, GE and SE echo planar images (EPI) was not severely distorted (Kording et al., 2014). In this study the distribution of vessel size was shown to be larger in untreated glioma mice when compared to glioma mice treated with anti-VEGF antibody (bevacizumab) (Kording et al., 2014).

### Blood oxygenation level dependent (BOLD) functional MRI

With the increase of SNR in small animal MRI it has become increasingly rewarding to advance upon investigations related to central nervous processing. Functional MRI (fMRI) in mice is a crucial tool to characterize transgenic mouse models of human cerebral pathologies and determine the outcome of therapies on the function of large-scale brain circuits. Functional MRI takes advantage of neurovascular coupling as surrogate of neuronal activity. Established measuring techniques track local temporal changes in either blood flow, blood volume or blood oxygenation—all shown as positively correlated with firing rates of adjacent neuronal cell populations (Kim and Ogawa, 2012). Most common techniques in human fMRI make use of the blood oxygenation level-dependent (BOLD) effect. Due to different magnetic properties of oxygenated and deoxygenated hemoglobin temporal changes in local field homogeneity can be detected by T_2_^*^ weighted MR techniques. Single shot gradient echo based echo planar imaging (GE-EPI) is commonly applied to meet the requirements on BOLD signal sensitivity and high temporal resolution.

**Figure 8:**
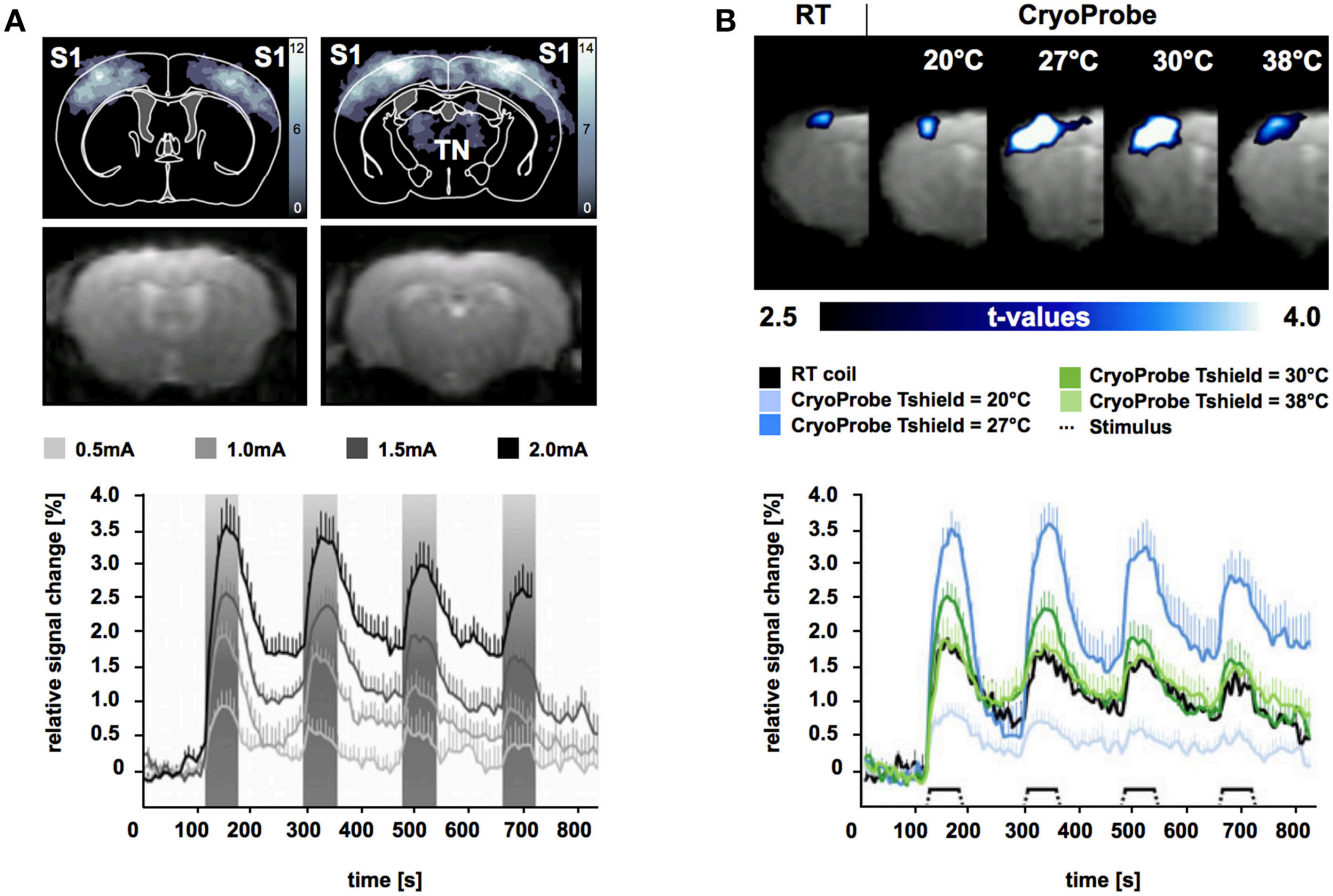
**Blood oxygenation level-dependent (BOLD) fMRI significance maps (upper panel) and signal intensity time courses (lower panel) in mice for electrical stimulation of the forepaw during isoflurane anesthesia (1%) and mechanical ventilation**. **(A)** Upper panel shows BOLD significance maps (upper row) for time series acquired using the CryoProbe revealing significant voxel clusters in primary somatosensory cortex (S1) and thalamic nuclei (TN) at 1.5 mA (*p* < 0.0001). Intensity corresponds to the number of animals displaying a significant BOLD signal. GE-EPI images (lower row) reveal only slight distortions. Lower panel shows signal intensity changes over time for significant voxel clusters in S1 during stimulation at 0.5, 1.0, 1.5, and 2.0 mA. Stimulus periods shaded in gray. From Bosshard et al. (2010), by permission of Wolters Kluwer Health, Inc. **(B)** Comparison of mouse fMRI data for significant voxel clusters in S1 acquired using a room temperature (RT) radiofrequency mouse head coil and the CryoCoil at different thermal shield temperatures. From Baltes et al. (2011), by permission of John Wiley & Sons Limited.

To gain sufficient SNR at short acquisition times of 1–8 s comes at the cost of spatial resolution limits in mouse fMRI. At (ultra)high magnetic fields partial brain volumes are commonly acquired with in plane resolutions of about 190–260 microns and a slice thickness of 500 μm (Adamczak et al., 2010; Baltes et al., 2011; Bosshard et al., 2012; Nasrallah et al., 2014; Schroeter et al., 2014). At these voxel sizes GE-EPI is prone to severe image distortions induced by macroscopic magnetic field inhomogeneities, which increase with field strength. Mostly affected regions are air-tissue boundaries (interfaces of bone, tissue, and air). Gaining SNR by using systems with B_0_≥11.7 T further increases EPI image distortions (Adamczak et al., 2010). Improving upon SNR by utilizing a CryoProbe permits preserving MR signal in imperiled brain regions. The CryoProbe was shown to achieve a three-fold increase in SNR for GE-EPI at 9.4 T compared to a conventional RT surface coil (Baltes et al., 2011). Leveraging on the sensitivity gain of a cryogenically cooled RF coil permits increases in receiver bandwidth, which facilitates inter-echo time shortening in EPI. This results in an increase in the bandwidth along the phase encoding direction which allows for reduction in EPI image distortion (Figure 8A).

Functional brain mapping surveys voxel-wise signal changes over time series of acquired image volumes. Signal and image consistency can be significantly impaired by physical (thermal noise, gradient heating) and physiological noise (cardiac, respiration, vascular oscillations). For mouse fMRI physiological noise is particularly relevant: due to the small body size inner organs like heart and lungs are in close proximity to the receiving RF head coil. Physiological noise (e.g., respiratory induced periodic changes in B_0_ field homogeneity) gets amplified with higher field strengths. Temporal SNR (tSNR) considers physical and physiological fluctuations over time making it an important measure for fMRI data quality. Baltes and colleagues reported a 1 to 3-fold increase in tSNR with the CryoProbe over a conventional RT mouse head coil at 9.4 T (Baltes et al., 2011).

Somatosensory studies using the CryoProbe revealed most significant BOLD signal magnitudes in mouse fMRI with external stimuli (Bosshard et al., 2010, 2012, 2015; Baltes et al., 2011; Mueggler et al., 2011; Schroeter et al., 2014). Signal intensity changes of up to 3.5% were observed in the primary somatosensory cortex for electro-stimulation (Figures 8A,B; Bosshard et al., 2010, 2012; Baltes et al., 2011; Schroeter et al., 2014) and moderate noxious heat stimulation (Bosshard et al., 2015) of the murine paw under isoflurane anesthesia. In murine fMRI studies investigating somatosensation and pain commonly anesthesia is applied for ethical and practical reasons (Borsook and Becerra, 2011). Under these conditions BOLD magnitude strongly depends on the applied anesthetic protocol and control of physiological parameters (Schroeter et al., 2014). This makes it somewhat difficult to compare fMRI data obtained with a CryoProbe setup to other somatosensory mouse fMRI studies using conventional room temperature RF coils.

A direct comparison of maximum BOLD signal changes during electrostimulation of the murine forepaw using the CryoProbe compared to a conventional RF mouse head coil was conducted by Baltes et al. (2011). In this study scalp temperature was found to be an important factor influencing the BOLD signal magnitude. To ensure physiological temperatures at the surface of the CryoProbe it features a thermal shield, which can be adjusted in a certain temperature range. An internal heater generates a thermal gradient along the ceramic surface. The shield heating can be adjusted to result in the desired temperature at the scalp-coil interface. In anesthetized mice reduced scalp temperatures promote vasoconstrictive effects ascertained by reduced baseline perfusion (Baltes et al., 2011). Higher SNR is expected to aid BOLD signal detection but should not alter the BOLD effect itself. At physiological temperatures (T_scalp_ of approx. 33.5°C) maximum BOLD magnitude was found to be similar for the CryoProbe and conventional RF mouse head coil during electrostimulation of the forepaw (Figure 8B). Mild cerebral hypothermia (T_shield_ = 27°C) was shown to significantly boost BOLD signal magnitude whereas severe hypothermia (T_shield_ = 20°C) decreased BOLD signal intensity, most likely due to impairments in physiological processes. The temporal noise for baseline measurements was significantly reduced for the CryoProbe for all thermal shield temperatures up to factor 2 compared to conventional RF mouse head coil (Baltes et al., 2011). Therefore, the CryoProbe provides higher BOLD signal sensitivity and might be capable to detect smaller neurovascular changes. The possibility to adjust its surface temperature permits to sustain more physiological thermal conditions in anesthetized mice.

## Cardiovascular applications

### *In vivo* assessment of cardiac morphology and function

Realizing that cardiovascular disease (CVD) is one of the leading causes of death (Heidenreich et al., 2011), research in pathologies related to heart function remain at the forefront of academia and pharmaceutical industry. For phenotyping of disease models (e.g., myocardial infarction or hypertension-induced myocardial injury/remodeling), as well as assessment of gender effects, evaluation of novel therapeutics and long-term follow-up studies non-invasive *in vivo* imaging of the heart is conceptually appealing. Cardiovascular magnetic resonance (CMR) is widely used in preclinical studies, the workhorse being an assessment of cardiac morphology and function. For a comprehensive and up-to-date review of CMR in small rodents please refer to Bakermans et al. (2015).

A highly reproducible quantitative assessment of cardiac morphology and function by CMR demands excellent spatial and temporal resolution. Both come at the cost of SNR loss, which can only be partially compensated for by increasing measurement time. Together with the small size of the mouse heart and its rapid motion (typical heart rates are 400–600 beats per minute) these SNR limitations pose serious challenges to CMR in mice. For cardiac chamber quantification and left ventricular function assessment an excellent delineation of myocardial borders, high ventricular blood-to-myocardium contrast and full coverage of the cardiac cycle with high temporal resolution are required. Furthermore, a complete coverage of the entire heart with a sufficiently high spatial resolution to facilitate reliable segmentation of the endo- and epicardial borders is crucial.

Current experimental CMR of mice uses dedicated birdcage volume RF coils or surface RF coil arrays with the geometry adjusted to the anatomy of the mice (Epstein, 2007; Heijman et al., 2007; Hiba et al., 2007; Young et al., 2009; Ratering et al., 2010; Schneider, 2011; Schneider et al., 2011) and affords images with a typical in-plane spatial resolution of 100–200 μm, heart coverage of 6–13 slices of 1.0 mm thickness and measurement of 10–20 cardiac phases (Epstein, 2007; Heijman et al., 2007, 2008; Hiba et al., 2007; Sosnovik et al., 2007; Young et al., 2009; Ratering et al., 2010; Bovens et al., 2011; Schneider, 2011; Schneider et al., 2011). Although providing acceptable image quality for the quantitative assessment of the left ventricle (LV), further improvement in image quality is highly desirable, particularly for the assessment of the right ventricle (RV). To meet this goal, enhancements in the spatial resolution are essential, which in turn build upon SNR improvements.

**Figure 9:**
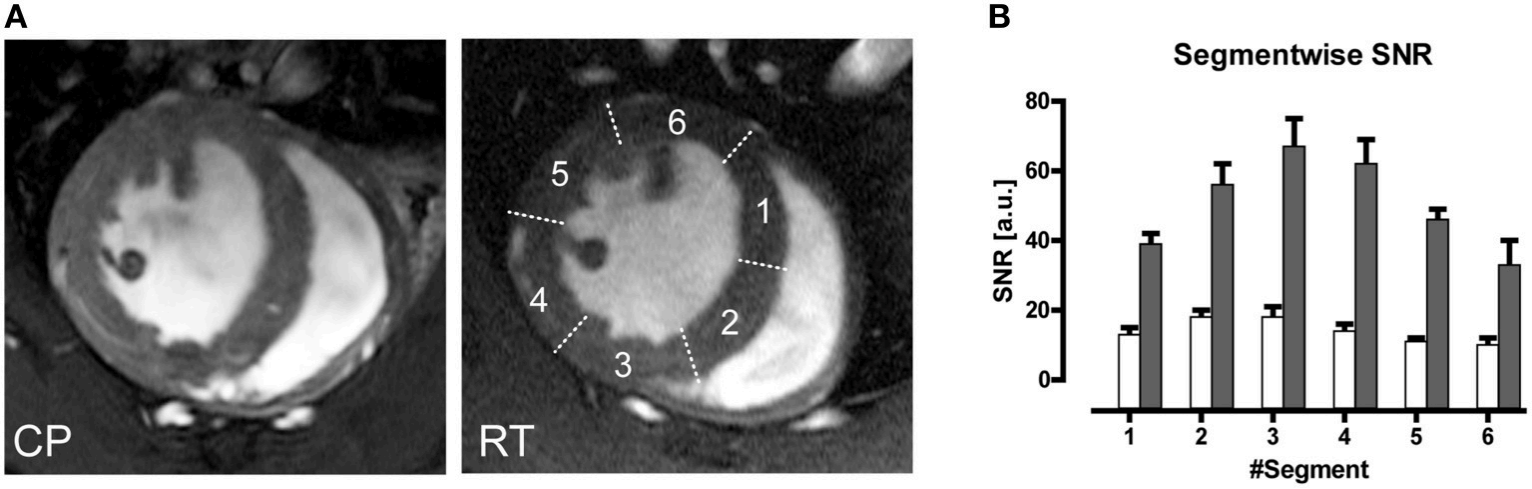
**(A)** Comparison of end-diastole short axis views acquired using a spatial resolution of (69 × 115 × 800) μm^3^. Myocardial border delineation and depiction of anatomic details for left ventricular papillary muscles and right ventricular trabeculae is enhanced in the CryoProbe (CP) image compared to the room temperature (RT) coil image. **(B)** Bar plot of mean left ventricular myocardium SNR measured in images acquired with the CryoProbe (gray bars) or RT RF coil (white bars) in the different segments of a six-segment model. The segments were numbered clockwise, starting at the inferoseptal segment. The region closest to the coil (segment 3) showed the highest SNR, and SNR decreased with distance from the coil. From Wagenhaus et al. (2012), by permission of Public Library of Science.

An average gain in SNR of 3.6 in murine cardiac MRI was achieved when comparing a CryoProbe at 9.4 T with a conventional room temperature RF coil setup (receive-only four-element mouse heart surface array RF coil in combination with a volume resonator for transmission (Wagenhaus et al., 2012). The SNR gain ranged 3.0–5.0 within the myocardium and could be translated into higher spatial resolution imaging. The highly detailed anatomical images derived from the CryoProbe acquisitions provided significantly improved myocardial border sharpness vs. the RT-coil acquisitions as illustrated in Figure 9A. Hard evidence for the benefit of the enhanced image quality was the improved reproducibility of the quantitative morphology and function assessment. Using the CryoProbe, intraobserver, and interobserver variability were smaller for almost all cardiac function parameters. For instance intraobserver variability in end-diastolic mass (EDM), end-diastolic volume (EDV), and end-systolic volume (ESV) were reduced on average by 59 and 66% for the left and right ventricle, respectively. The practical advantage is two-fold. An increased sensitivity will allow an earlier detection of pathological changes as well as a more thorough assessment of therapeutic effects. A smaller statistical variability within experimental groups allows for a significant reduction in animal group size.

Currently available cryogenic RF technology comes along with some challenges for performing CMR. The CryoProbe cannot easily be placed onto any anatomical region of interest since it must be installed inside the magnet bore. Instead, the animal is placed underneath the surface RF coil using a dedicated cradle. This set-up is tailored and well-suited for mouse brain MRI. To use it for CMR requires a special supine positioning of the mouse. Training and practice is necessary to position the heart correctly within the field of view of the CryoProbe. The supine position alters the shape of the RV (see short-axis view in Figure 9A and long-axis view in Wagenhaus et al., 2012) but does not impact on the functional parameters (Wagenhaus et al., 2012). In some animals the supine position is accompanied by a difference in motion of the septum, which continues throughout diastole and results in blurring of the endocardial borders of the septum.

Inherent to surface coils is a decrease in signal-amplitude with increasing distance (as explained above). Hence the CryoProbe setup—just like the conventional RT surface RF coil setup—displays a spatial variation in SNR across the myocardial segments of a short axis view (Figure 9B), which can be explained with the proximity to the surface coil. Inhomogeneity on the RF transmit side (${\text{B}}_{1}^{+}$) is usually of little concern for gradient-echo based protocols such as the CINE FLASH commonly used for assessment of cardiac morphology and function (Figure 9A; Wagenhaus et al., 2012). Yet for techniques in which an exact flip angle is crucial—such as saturation based T_1_-weighted gadolinium-enhanced first pass bolus perfusion studies (Utz et al., 2007, 2008) or inversion recovery prepared T_1_-mapping for detection of fibrosis or characterization of myocardial tissue (Messroghli et al., 2003)—${\text{B}}_{1}^{+}$ inhomogeneity may be an unfavorable characteristic of the CryoProbe. Such cardiac applications can benefit from using a volume resonator for transmission in conjunction with the state-of-the-art receive-only CryoProbe.

In conclusion, cryogenically cooled RF coils represent a valuable means of enhancing the image quality in *in-vivo* CMR of mice. They permit high spatial resolution CINE imaging of the mouse heart with excellent SNR, which measurably improves the reproducibility of quantitative cardiac morphology and function assessment. This is important since segmentation of the myocardium is challenging and prone to introduce significant data variability.

### *Ex vivo* MR microscopy of the rodent heart

MR microscopy is a powerful tool to get an insight into the micro architecture of myocardial tissue and provides results similar to histology in a non-invasive matter. High spatial resolution and an appropriate organ coverage are both essential. Quantitative techniques like T_2_^*^ mapping, diffusion tensor imaging (DTI) or quantitative susceptibility mapping (QSM) can complement the information provided from common MR contrasts and allow intersubject assessment. DTI or QSM require 3D datasets with high isotropic resolution. Combining the requirements of high resolution MR microscopy with large volume or whole heart spatial coverage already puts a strain on balancing SNR with scan time. T_2_^*^ weighting and diffusion weighting, as needed for DTI or QSM, are also factors that inherently reduce SNR: Sufficient initial SNR is needed, to ensure adequate signal even at long TEs or for strong diffusion weighting. Thus, several features of quantitative myocardial MR microscopy constrain SNR and bring about the need for lengthy scan times to compensate for SNR losses. Even in *ex vivo* scans where acquisition time is by far not as limited as in *in vivo* scans, counteracting SNR losses by averaging can easily result in days of acquisition time. Unfortunately, this condition is not feasible even in an *ex vivo* setting. Using a cryogenic RF probe helps to balance the competing constraints of high spatial resolution, high spatial coverage and sufficient T_2_^*^ or diffusion weighting in acceptable acquisition times at ample signal to noise.

**Figure 10:**
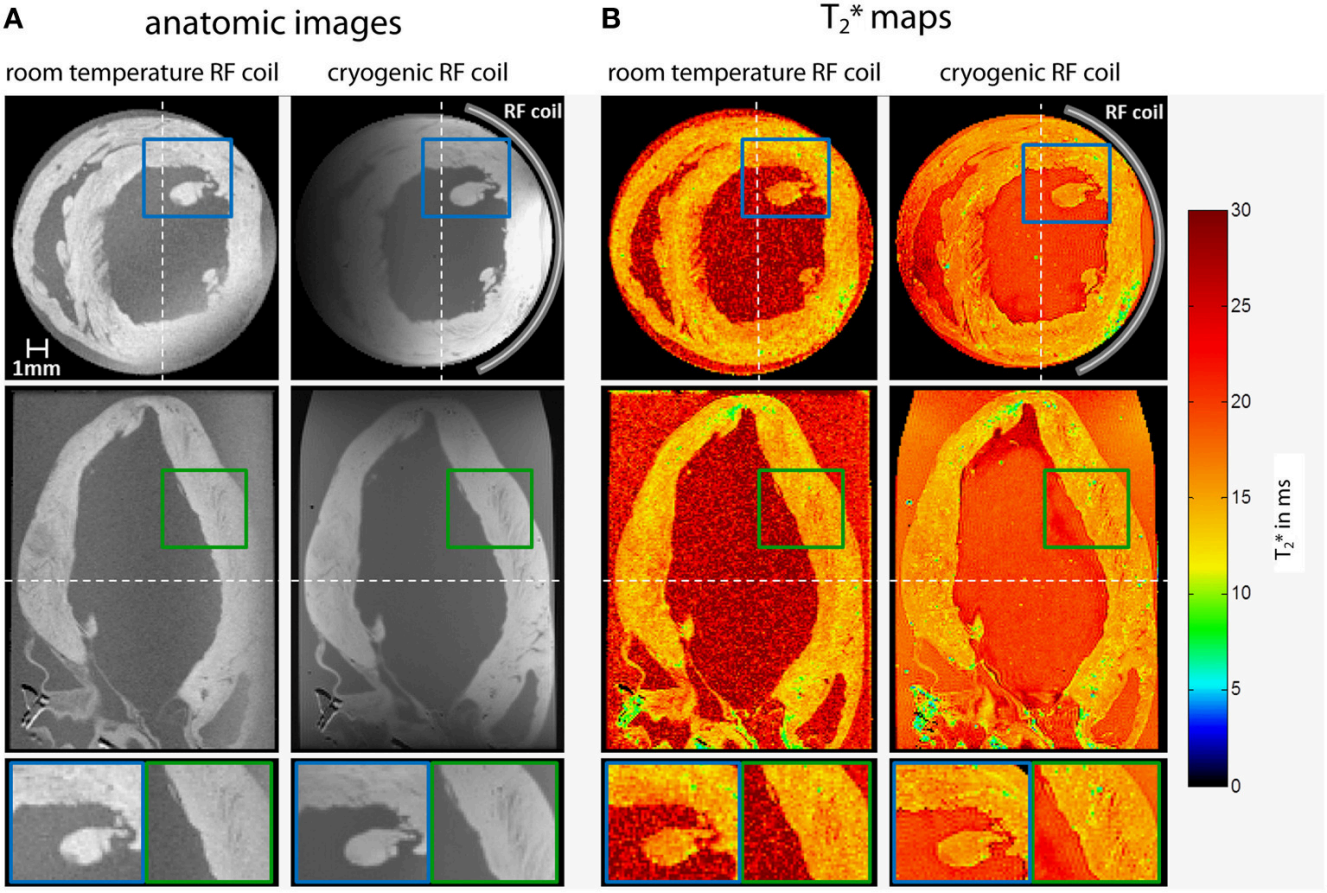
Axial (top) and coronal (center) views of high resolution T_2_* weighted 3D MR microscopy images (A) and corresponding T_2_* maps (B) of the fixed *ex vivo* rat heart acquired using a room temperature volume RF resonator and a cryogenic surface RF probe at 9.4 T [3D multi echo gradient echo technique, TR = 19 ms, TE = 2.14–13.54 ms, TE spacing 2.85 ms, spatial resolution = (94 × 94 × 94) μm^3^, FOV = (20 × 15 × 15) mm^2^, acquisition time 12.7 h]. Bottom: Detail views of areas marked by colored rectangles. Dashed white lines mark the position of the slices. Data from Huelnhagen et al. (2014); Peper et al. (2015).

**Figure 11:**
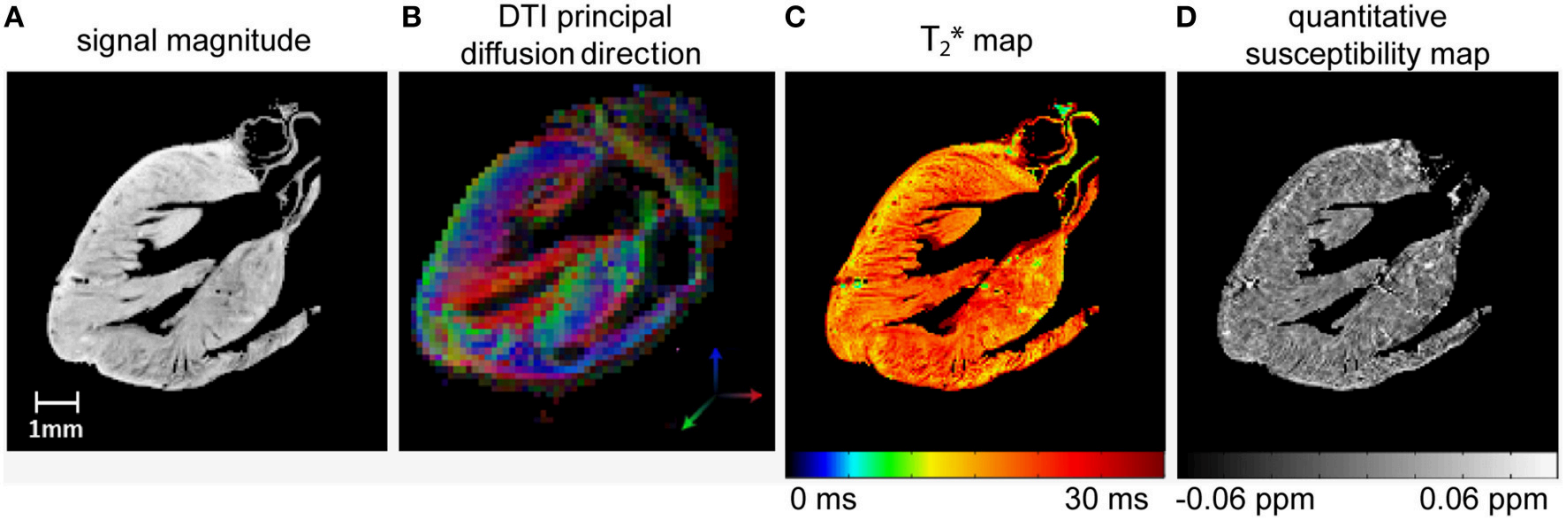
Sagittal slices of high resolution 3D MR microscopy images of the fixed *ex vivo* mouse heart acquired at 9.4 T using a cryogenic RF probe: (A) signal magnitude image, TE = 2.14 ms (B) principal diffusion direction derived from DTI (C) T_2_* map and (D) quantitative susceptibility map. Acquisition parameters: 3D multi echo GRE: TR = 250 ms, TE = 2.14–44.96 ms, TE spacing 2.85 ms, spatial resolution = (55 × 55 × 55) μm^3^, FOV = (10 × 10 × 10) mm^3^, TA = 13.7 h; DTI-EPI: TR = 1000 ms, TE = 22.84 ms, spatial resolution = (156 × 156 × 156) μm^3^, FOV = (10 × 10 × 10) mm^3^, b = 2000 s/mm^2^, 30 directions, TA = 17 h. Data from Peper et al. (2015).

Figure 10 shows examples for high resolution T_2_^*^ weighted 3D MR microscopy of the fixed *ex vivo* rat heart at 9.4 T. These short and long axis views of the heart were derived from acquisitions using a conventional room temperature birdcage RF coil and a cryogenic surface RF coil (Huelnhagen et al., 2014; Peper et al., 2015). Signal magnitude and corresponding T_2_^*^ maps derived from fitting the signal decay over 5 echo times are shown. Sub-millimeter structures, which can clearly be delineated in images obtained with the CryoProbe, are dominated by noise in the images acquired using the room temperature RF coil. With identical sequence and protocol settings and equal acquisition times approximately a five-fold mean SNR enhancement was achieved in this example when using the cryogenic RF probe. Attributing the SNR increase in this case solely to the fact that a cryogenic probe has been used would not be entirely appropriate since moving from a volume to a surface RF resonator usually goes along with an SNR gain at least in regions close to the RF coil. Notwithstanding this limitation it is fair to state that high SNR level images obtained with the cryogenic RF probe cannot be accomplished with the room temperature probe using the same imaging protocol and scan times. The SNR and signal uniformity implications of the limited field of view and B_1_ gradient of the cryogenic surface RF coil can be recognized from the axial images. These non-uniformities can be corrected but might constitute a challenge for sample sizes not matching the coil geometry. If the sample size however, is in the range of the field of view specifications of the cryogenic RF surface coil, high resolution MR microscopy provides images that are comparable with histology, earning heart MR microscopy the moniker of cardiac MR histology. Figure 11 illustrates an example of a fixed *ex-vivo* mouse heart scanned in 3D with an isotropic spatial resolution of 55 μm^3^ using a cryogenic surface RF coil at 9.4 T (Peper et al., 2015). Making use of the SNR gain of the cryogenic RF probe, despite the very high spatial resolution, an SNR of more than 60 was achieved for the first echo (TE = 2.14 ms). Even for long echo times SNR obtained with the cryogenic RF coil is appropriate to accommodate accurate T_2_^*^ mapping.

To summarize, MR microscopy of the heart with a high spatial coverage heavily benefits from using cryogenic RF resonators especially when contrast weighting is required, as in parametric mapping or diffusion weighted applications. The benefit is paramount during the characterization and phenotyping of animal models of cardiac disease as it provides a valuable means of getting a better understanding of myocardial microstructure and myocardial remodeling. The value of such improvements are in positive alignment with human MR studies at ultrahigh magnetic fields (Von Knobelsdorff-Brenkenhoff et al., 2010, 2013; Dieringer et al., 2011; Thalhammer et al., 2012; Winter et al., 2012; Graessl et al., 2014) which strive for a relative spatial resolution (number of pixel per cardiac anatomy) approaching that of experimental MRI in small rodents with the ultimate goal to provide imaging means for diagnostics and for guiding treatment decisions in cardiovascular and metabolic diseases (Niendorf et al., 2010, 2013, 2015a).

## Renal applications

### Ex vivo MR microscopy of the rodent kidney

**Figure 12:**
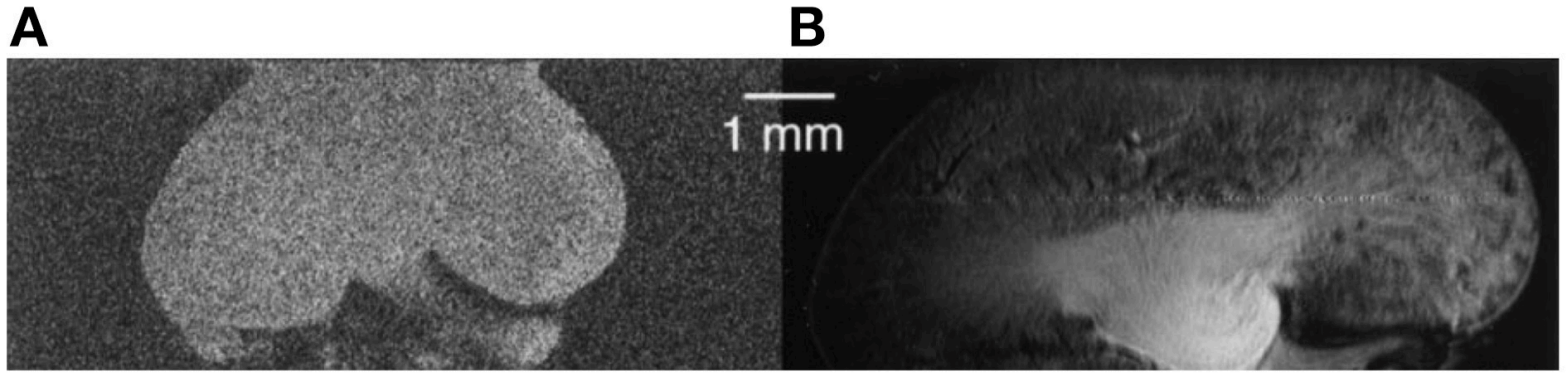
**Early proof-of-concept for renal MR microscopy at 9.4 T with 17 μm in-plane resolution. (A)** Room temperature Helmholtz RF coil. **(B)** High-temperature superconducting Helmholtz RF probe. From Hurlston et al. (1999) by permission of John Wiley & Sons Limited.

Hurlston et al. (1999) ventured into an early exploration of *ex vivo* mouse kidney MR microscopy; their prototype HTS Helmholtz RF probe provided a seven-fold gain in SNR compared to a room temperature Helmholtz RF coil. Using the superconducting coil, the feasibility of renal MR microscopy was demonstrated at 9.4 T with 17 μm in-plane resolution in little more than an hour scan time (Figure 12; 3D spin-echo, (17 × 17 × 136) μm^3^ spatial resolution).

The sensitivity gain of cryogenic RF coil technology can be put to good use for renal MR microscopy of glomeruli, the microscopic filtering units of the kidney. Glomerular size and number is thought to be linked to several renal and cardiovascular diseases (Hoy et al., 2008). While established techniques for measuring/counting glomeruli rely on extrapolations made from measurements/counts within small samples (Beeman et al., 2011), a total and direct quantification can be made by renal MR microscopy (Beeman et al., 2011; Heilmann et al., 2012). By exploiting the sensitivity boost of a CryoProbe, Heilmann et al. (2012) were able to quantify glomerular number and size in ferritin labeled rat kidneys *ex vivo* with a scan of less than 5 h duration. Automatic segmentation of 3D GRE images with (35 × 35 × 35) μm^3^ spatial resolution yielded glomerular diameters (mean ± SD = 109 ± 4.9 μm) and counts (mean ± SD = 32,785 ± 3117) with low intra-subject variability in one-seventh of the time required for traditional stereology.

### *In vivo* structural and functional renal MRI

**Figure 13:**
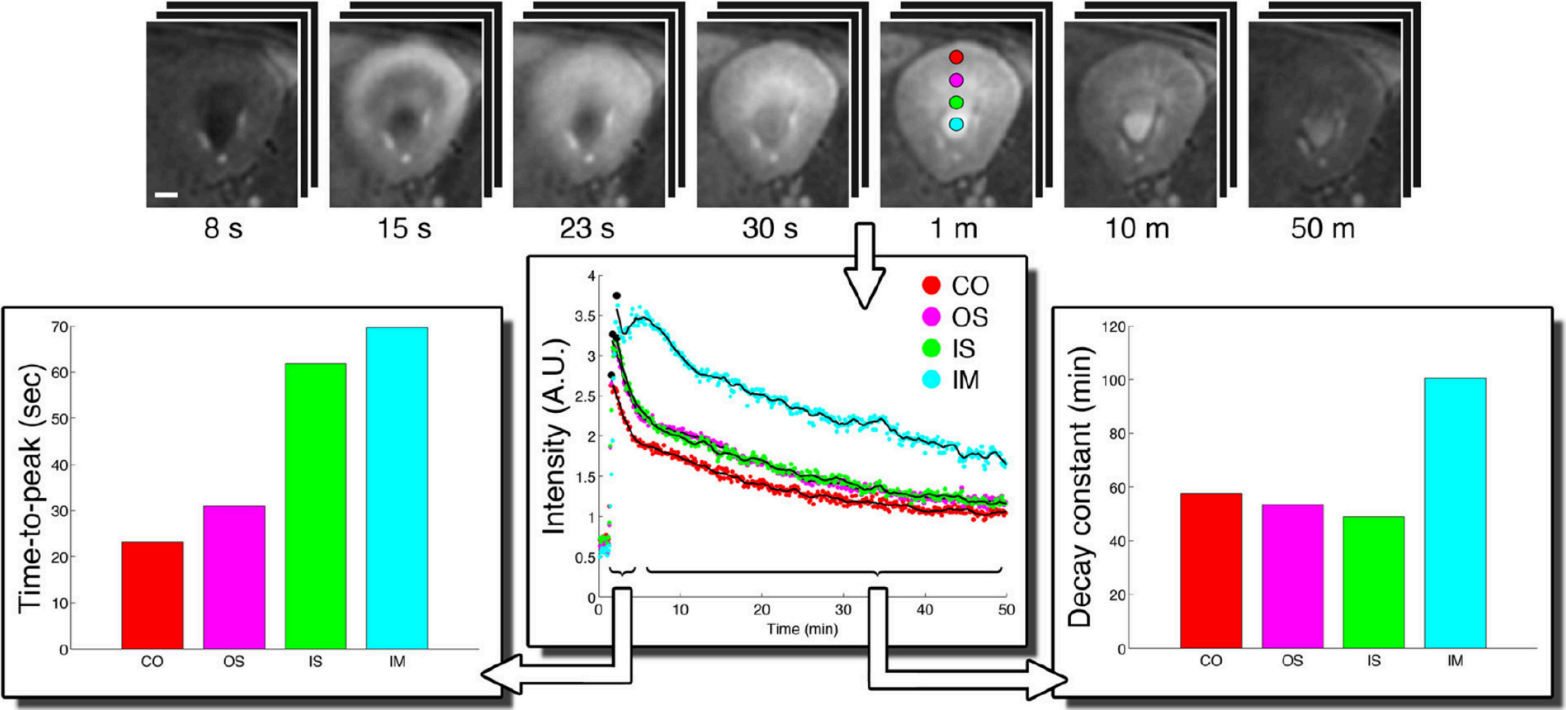
**CryoProbe application in fast DCE-MRI of the mouse kidney (Xie et al., 2014)**. T1-weighted renal images (top) are shown for 7 out of 390 acquired time points. Signal time-courses for small regions-of-interest in renal cortex (CO), outer stripe (OS), and inner stripe (IS) of the outer medulla, and the inner medulla (IM) illustrate the excellent SNR. The derived parameters time-to-peak and decay constant reflect the inter-layer functional differences. From Xie et al. (2014) by permission of John Wiley & Sons Limited.

**Figure 14:**
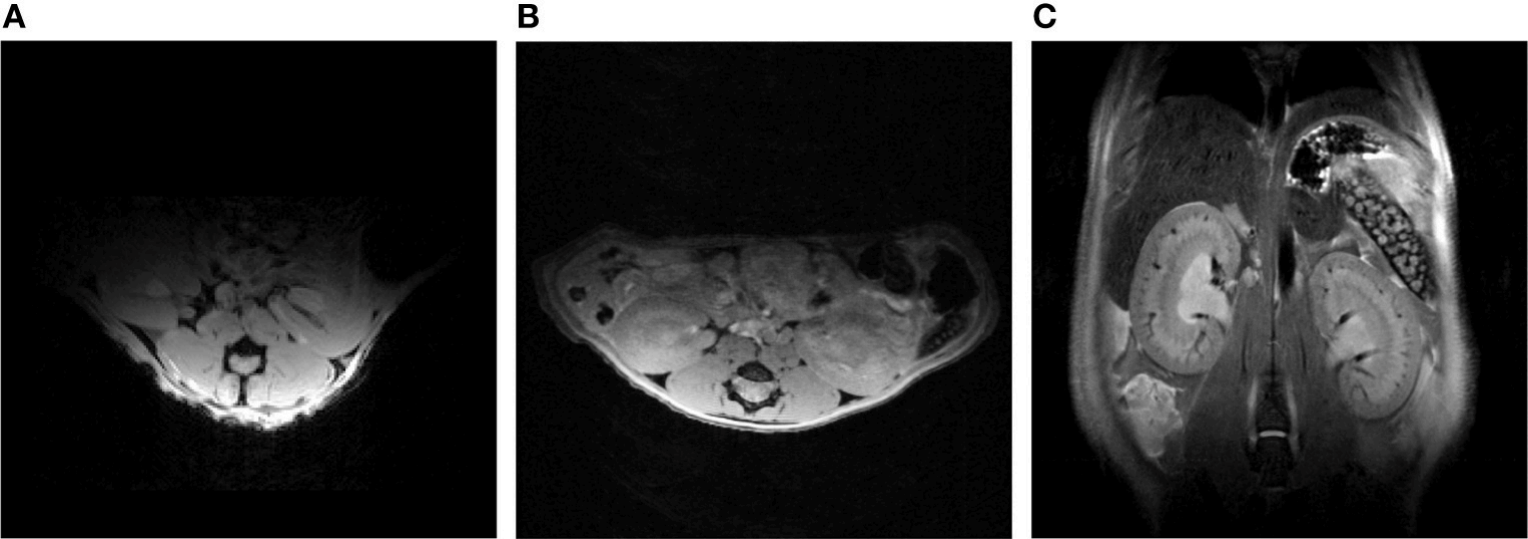
**Comparison between an axial view of the mouse kidneys using a mouse CryoProbe and a rat CryoProbe at 9.4 Tesla**. The mouse CryoProbe only permits imaging of one kidney due to the limited field of view **(A)**. Using the rat CryoProbe enables simultaneous imaging of both mouse kidneys with a more homogenous signal intensity distribution (**B**: axial view, **C**: coronal view). Experimental details are: **(A)** FLASH, TR = 350 ms, TE = 5.4 ms, FOV = (30 × 20) mm^2^, resolution = (156 × 208) μm^2^, slice thickness = 1000 μm, scan time = 0.6 min. **(B)** FLASH, TR = 120 ms, TE = 2.9 ms, FOV = (30 × 30) mm^2^, resolution = (136 × 94) μm^2^, slice thickness = 500 μm, scan time = 1.3 min. **(C)** TurboRARE, TR = 750 ms, TE = 24 ms, FOV = (30 × 30) mm^2^, resolution = (94 × 94) μm^2^, slice thickness = 500 μm, scan time = 4.5 min.

To date the potential for improving spatial resolution and increasing temporal resolution by use of a CryoProbe for *in vivo* renal MRI remains largely untapped. The boost in SNR can be expected to be similar to that reported for cardiac applications, i.e., approximately three-fold (Wagenhaus et al., 2012). Organ size and depth of location within the thorax/abdomen are comparable. The same room temperature RF coils are typically used for both applications, commonly a four-element saddle-shaped receiver (RX) array in conjunction with a larger transmit (TX) volume resonator. *In vivo* MRI of a mouse kidney is perfectly feasible with a mouse CryoProbe (Xie et al., 2014; Figure 13). However, imaging of both kidneys simultaneously requires a much larger field of view than needed for a single kidney or the heart. The rat CryoProbe may be an attractive alternative as it provides a uniform signal intensity distribution over a larger field of view than the mouse CryoProbe (Figure 14). Objective signal and noise measurements will however still be required since these coils were not originally designed for matching the geometry of the mouse body and for optimized kidney imaging.

The combination of high speed acquisition with almost microscopic spatial resolution is of great interest also in other areas of functional renal MRI, such as T_2_^*^ monitoring, as surrogate for renal blood oxygenation. Parametric mapping of T_2_^*^ with sub-minute temporal resolution is essential for capturing the fast dynamic changes in renal hemodynamics and oxygenation during acute ischemic events or physiological test stimuli (Pohlmann et al., 2013b, 2014a). Comparison with renal MR microscopy images (Xie et al., 2012; Niendorf et al., 2015b) suggests that the limited spatial resolution of current *in vivo* protocols used for monitoring T_2_^*^ masks some of the underlying intra-layer heterogeneity of renal structure and function. Higher spatial resolution for renal parametric MRI, which puts a strain on SNR, is therefore warranted especially in the mouse. Reduced oxygenation of intrarenal blood can lead to an additional loss of signal, which can be substantial in images acquired at larger echo times. Elucidating ischemia/reperfusion (I/R) injury, a consequence of kidney hypoperfusion or temporary interruption of blood flow—a common cause of acute kidney injury (AKI)—requires fast continuous T_2_^*^/T_2_ mapping throughout baseline, ischemia, early reperfusion, and recovery (Pohlmann et al., 2013b) and hence benefits from scan acceleration using parallel imaging strategies. To this end, the SNR gain offered by a multi-channel receive cryogenic RF coil array is instrumental for compensating noise amplification inherent to parallel imaging (Niendorf and Sodickson, 2006a,b, 2008). The speed gain is of benefit in preclinical renal MRI studies where multiple parameters are assessed by multiple modalities including (i) quantitative physiological measurements such as renal perfusion pressure, renal blood flow, local cortical and medullary tissue pO_2_ and blood flux and, (ii) comprehensive MRI protocols with tight spatio-temporal resolution constraints dictated by renal (patho)physiology and the interleaving with the quantitative physiological measurements (Pohlmann et al., 2014a). For all these reasons, cryogenic RF coil technology holds great potential for enabling non-invasive *in vivo* investigations into renal hemodynamics and oxygenation with MRI. Conventional methods for assessing renal hemodynamics and oxygenation such as fiber-optical pO_2_ probes or ultrasonic flow probes provide quantitative physiological data, but are invasive and the fiber probes enable measurements in pin-point locations only (Arakelyan et al., 2013; Pohlmann et al., 2013a).

### Assessing renal perfusion, oxygenation, or inflammatory cell infiltration using fluorine (^19^F) MRI

Renal tissue hypoxia and inflammatory mechanisms play prominent roles in the pathophysiological chain of events that lead to acute kidney injury and promotes progression from acute injury to chronic kidney disease. Dependent on disease etiology, the contribution and impact of inflammatory mechanisms may vary (Chawla et al., 2014). Inflammatory processes, including early responses dominated by the innate immune system as well as later responses by the adaptive immune system, can support repair and restoration of renal functions but may also promote renal tissue injury and transition to chronic disease (Bonventre and Yang, 2011; Kinsey and Okusa, 2014; Molitoris, 2014).

Acute kidney injury can also trigger a systemic immune response that may lead to secondary dysfunction of organs such as heart, brain, and lung (Grams and Rabb, 2012). Alternatively, systemic immune diseases can have a crucial impact on the kidney e.g., antineutrophil cytoplasmic antibody (ANCA)-associated vasculitis often affects the kidneys leading to rapid-progressive glomerulonephritis (Schreiber and Choi, 2015).

Renal blood volume, blood oxygenation, and inflammatory cell migration can be assessed and monitored using cutting-edge ^19^F MR techniques (Ruiz-Cabello et al., 2011; Hu et al., 2014). MRI of x-nuclei suffers from an inherently low SNR due to limited MR sensitivity (^19^F ≈ ^1^H) but also a low abundance of nuclei in the tissue of interest. Indeed, ^19^F is virtually absent in the living tissues of rodents and the ^19^F signal is created by injection of exogenous ^19^F contrast agents. A commonly used variant of such ^19^F agents is perfluorocarbon (PFC) (Ruiz-Cabello et al., 2011), which can be used to label inflammatory cells *in vivo* after systemic application (Waiczies et al., 2013) or track specific immune cells *in vivo* after *in vitro* labeling (Ahrens et al., 2005; Waiczies et al., 2011).

**Figure 15:**
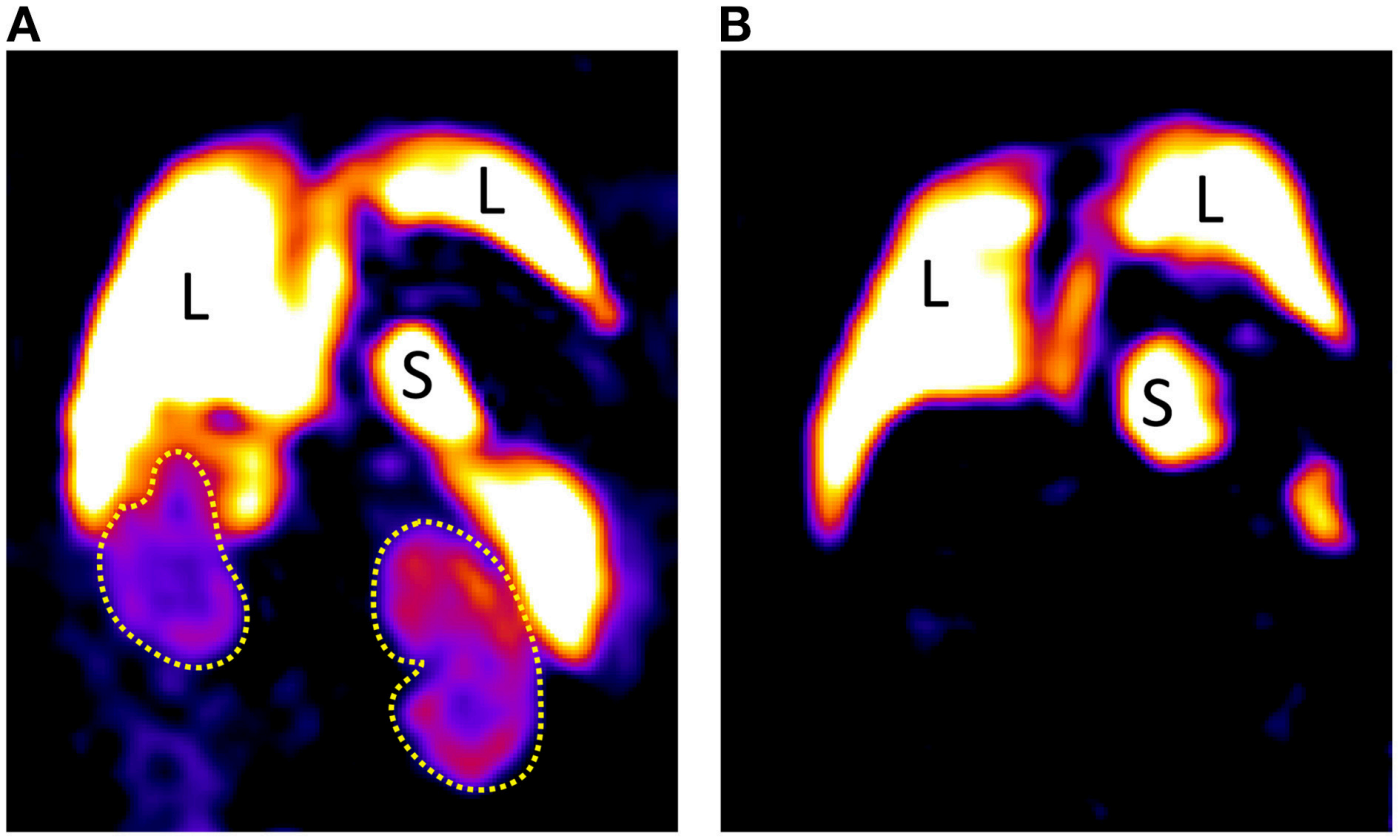
**(A)** Coronal ^19^F images of a mouse with ANCA-induced glomerulonephritis; 8 weeks after bone marrow transplantation showing extensive renal inflammatory cell infiltration, particularly in the cortex in comparison with a naïve mouse **(B)** showing a high signal in liver and spleen, but none in the kidneys. The low SNR of room temperature RF technology severely limits the spatial resolution achievable even at extended scan durations of almost 1 h. L indicates liver, S indicates spleen, and yellow dot lines depict kidneys. Data from Schreiber et al. (2013).

Systemic i.v. administration of ^19^F nanoparticles (droplet emulsion) results in uptake and self-labeling by phagocytic immune cells e.g., macrophages, neutrophils or dendritic cells. Application of these nanoparticles to an ANCA-induced glomerulonephritis model demonstrated a significant ^19^F MRI signal in the kidney (in contrast to kidneys of control animals that showed negligible signal). This method provides an *in vivo* depiction of renal inflammatory cell dynamics (Figure 15; Pohlmann et al., 2014b).

Yet, the rather low spatial resolution of (0.94 × 0.94 × 1.88) mm^3^ and required acquisition time of almost an hour leave a lot to be desired. Here the low SNR is a fundamental factor that is restricting the capabilities of ^19^F MR in rodents. SNR negatively correlates to the detection limit for ^19^F labeled cells. Applications in which harvested immune cells are labeled *in vitro* and re-applied to an animal have an even greater need for high sensitivity because in this case the number of labeled cells in the region of interest is much smaller (Waiczies et al., 2013).

Noteworthy for ^19^F MRI, a gain in SNR of 3.0–3.5 for the ^13^C CryoProbe (Sack et al., 2014) and 2.5 for the ^1^H CryoProbe (Baltes et al., 2009) were reported at 9.4 T. Considering the close proximity of ^19^F and ^1^H frequency, an SNR gain of at least 2.5 will be expected for a ^19^F CryoProbe at 9.4 T, a gain that would permit an enhanced spatio-temporal resolution of preclinical ^19^F MRI and pave the way for better detection and more efficient tracking of cellular therapies such as dendritic cells that are used in a variety of cancers. With a technology that provides better detection of cells, even in far reaching organs, the best cell therapy solutions for pathologies such as recalcitrant cancers can be determined more efficiently and swiftly. These therapeutic solutions in combination with advanced clinical imaging technologies can ultimately be translated to the clinic and incorporated into personalized medicine.

## Outlook

The cryogenic RF coil technology has introduced significant advancements to small animal MR image acquisition in different areas of disease research. With the ever increasing clinical needs and research explorations looming over the MR horizon, further innovations in association with this technology are nevertheless likely to be expected. The opportunity of increasing sensitivity as well as spectral, spatial, and temporal resolution as a result of increased SNR with the cryogenic RF coil technology opens up more prospects for advancing evolving MR techniques in spectroscopy (MRS), imaging (MRI), or spectroscopic imaging (MRSI). MR methods for studying the detection and distribution of metabolites containing proton (^1^H) and x-nuclei e.g., carbon (^13^C), oxygen (^17^O), sodium (^23^Na), and phosphorous (^31^P) will benefit immensely from the introduction of cryogenically-cooled coils. In addition to the increased SNR, which is particularly favorable for nuclei with a relatively low biological abundance (e.g., ^13^C, ^23^Na, ^31^P), better spectral resolutions are expected to reveal metabolites that have until now remained undetected, keeping in mind that resolution comes at a cost in SNR. Another evolving field that will benefit from cryogenically cooled RF coils is functional MRS (fMRS), which when used in conjunction with BOLD fMRI will be invaluable to study changes in resting and activation states in the brain. MRSI employing high-speed echo-planar encoding will also benefit from sensitivity gains to spatially map multiple tissue metabolite signals *in vivo*.

A gain in sensitivity and resolution that can be delivered by cryogenically cooled RF coils can be attained by increasing magnetic field (B_0_) strengths, but the side effects and costs associated with higher B_0_ are in such a case non-existent. Challenging adverse effects of higher B_0_ (that can be eliminated with cryogenic RF coil cooling at lower fields) include saturation-related signal losses due to longer T1 relaxation times, shorter echo time acquisitions to compensate for sensitivity losses due to shorter T_2_ relaxation times and line broadening or off-resonant effects due to B_0_ susceptibility effects. For small animal imaging, cryogenically cooled RF coils would benefit from small cryogen-liquid free magnets systems with variable field operation and automatic field ramping. These MR systems can be operated at magnetic field strengths of 1.5, 3.0, or 7.0 T and hence provide opportunities for harmonizing basic with translational research. The installation and maintenance of such MR systems is affordable and thus ideal for preclinical research. Equipped with one cryogenically cooled RF coil such MR systems with cryogenic liquid-free magnets will be an invaluable technology for disease phenotyping, multi-modal imaging as well as for spanning of an assortment of x-nuclei for metabolic, molecular, and cellular imaging.

The benefits of the cryogenically-cooled RF coil technologies are in positive alignment with our human MR studies at ultrahigh B_0_ fields, which strive for the spatial resolution used in experimental small animal MRI, but necessitate complex techniques (such as volume-selective higher-order B_0_ shimming and phase correction strategies) to counterbalance the B_0_ adverse effects. Considering the advantages of cryogenically cooled RF coils and the expected immense application for human studies a swift clinical translation is warranted. For starters, tremendous sensitivity gains will be expected when using smaller cryogenically cooled coils in association with higher B_0_; the stronger magnetic coupling between small coil and sample is attributed to higher signal sensitivities and the smaller volume of tissue seen by smaller coils is attributed to lower noise. MR applications that will particularly benefit from cryogenically-cooled RF coil systems, particularly at UHF strengths, are diffusion weighted imaging (DWI) methods employing stronger diffusion-weighting (*b*-values > 2000 s/mm^−2^) in order to demonstrate the biexponential decay of brain diffusion, and better differentiate between different water compartments, especially during ischaemic and other brain pathology (Norris and Niendorf, 1995; Dijkhuizen et al., 1996; Niendorf et al., 1996). Although higher *b*-values yield higher diffusion contrast, lower SNR is expected on heavily diffusion-weighted images, such that cryogenically-cooled coils will surely be invaluable for this application. Alternatively, the SNR gain inherent to cryogenically-cooled RF coils could be transferred into using a larger range of diffusion weighting, which would be of great benefit for *in vivo* explorations into the biophysics of water diffusion in tissue (Niendorf et al., 1994; Norris et al., 1994; Pyatigorskaya et al., 2014; Le Bihan and Iima, 2015) but also for the study of intracellular compartmentation of metabolites, with the implication feeding into neuroradiology, neurology and related clinical disciplines (Najac et al., 2014). Other foreseen human applications for cryogenically-cooled RF coils will include anatomic micro-imaging of the skin. Pioneering work undertaken with a HTS coil at 1.5 T recently demonstrated a subnanoliter spatial resolution (80 μm^3^) for human skin MR microscopy (Laistler et al., 2015). Skin-sodium storage, as a physiologically important regulatory mechanism for blood pressure, volume regulation, and indeed survival, has recently been rediscovered. This prompted the development of MRI methods to assess sodium storage in humans with ^23^Na-MRI at 3.0 Tesla (Kopp et al., 2012, 2013) and at 7.0 T; the latter facilitating a spatial resolution across the skin of as good as 1 mm in-plane (Linz et al., 2015). To this end, cryogenically cooled RF technology indeed represents a powerful research tool that can potentially help to unlock questions regarding Na^+^ balance and Na^+^ storage functions of skin with the ultimate goal to provide imaging means for diagnostics and for guiding treatment decisions in cardiovascular and metabolic diseases. It is however envisioned that the benefits of cryogenically-cooled coils for human application at ultrahigh fields will also go beyond skin microimaging of sub-nanoliter voxels. The significance of the cryogenic technology to advance upon current x-nuclei MR methods for metabolic probing (Linz et al., 2015) and pharmacological studies (Ji et al., 2015) is an area that will definitely receive considerable attention in the coming years.

## Author contributions

TN, AP, and SW wrote the manuscript with help from HR, TH, and MK. HW, EP, ES, AS, RK, and KS contributed to data and intellectual feedback.

## Grant sponsors

This work was supported (in part, TH) by the DZHK (German Centre for Cardiovascular Research) and by the BMBF (German Ministry of Education and Research. HW received support by the German Federal Ministry of Education and Research (KMU-innovativ- Medizintechnik, MED-373-046). AP and HR were supported by the Helmholtz Alliance HGF/HMGU (Imaging and Curing Environmental Metabolic Diseases, HA-314).

### Conflict of interest statement

Sonia Waiczies received research grants from Novartis for a different project. Helmar Waiczies is employed by and Thoralf Niendorf is founder of MRI.TOOLS GmbH. Klaus Strobel is employed by Bruker BioSpin. No conflicts of interest were disclosed by the other authors.
